# Diverse roles of pontine NPS-expressing neurons in sleep regulation

**DOI:** 10.1073/pnas.2320276121

**Published:** 2024-02-21

**Authors:** Lijuan Xing, Xianlin Zou, Chen Yin, John M. Webb, Guangsen Shi, Louis J. Ptáček, Ying-Hui Fu

**Affiliations:** ^a^Department of Neurology, University of California San Francisco, San Francisco, CA 94143; ^b^Zhongshan Institute for Drug Discovery, Shanghai Institute of Materia Medica, Chinese Academy of Sciences, Zhongshan 528400, China; ^c^Department of Neurology, Weill Institute for Neurosciences, University of California San Francisco, San Francisco, CA 94143; ^d^Kavli Institute for Fundamental Neuroscience, University of California San Francisco, San Francisco, CA 94143; ^e^Institute of Human Genetics, University of California San Francisco, San Francisco, CA 94143

**Keywords:** NPS, parabrachial nucleus, central gray of the pons, sleep, neural circuitry

## Abstract

Neuropeptide S (NPS) was postulated to be a wake-promoting neuropeptide with unknown mechanism, and a mutation in its receptor (NPSR1) causes the short sleep duration trait in humans. We investigated the role of different NPS^+^ nuclei in sleep/wake regulation. Loss-of-function and chemogenetic studies revealed that NPS^+^ neurons in the parabrachial nucleus (PB) are wake-promoting, whereas peri-locus coeruleus (peri-LC) NPS^+^ neurons are not important for sleep/wake modulation. Further, we found that a NPS^+^ nucleus in the central gray of the pons (CGPn) strongly promotes sleep. Fiber photometry recordings showed that NPS^+^ neurons are wake-active in the CGPn and wake/REM-sleep active in the PB and peri-LC. Blocking NPS–NPSR1 signaling or knockdown of *Nps* supported the function of the NPS–NPSR1 pathway in sleep/wake regulation. Together, these results reveal that NPS and NPS^+^ neurons play dichotomous roles in sleep/wake regulation at both the molecular and circuit levels.

## Significance

We previously identified a mutation in the only receptor for the neuropeptide S (NPS), NPSR1 (NPS receptor 1), that enables people and mice to have reduced sleep duration. NPS is an important neurotransmitter that has been shown to play roles in regulating arousal, mood, food intake, and memory. Since the neuro-network involved in sleep/wake behavior is expected to be complex, we set out to investigate the role of NPS in various brain nuclei as a step toward further mapping the network. We found that NPS neurons in three different nuclei are either wake-promoting, sleep-promoting, or without effect, highlighting the intricate complexity of the sleep/wake-modulating network.

Many neurons and circuits relevant to sleep/wake modulation have been established by coupling their activities with sleep/wake states or by their connections with previously identified sleep centers (1–3). Alternatively, sleep/wake neurons and circuits can be revealed by studying sleep genes identified through genetic approaches (4–17).

Neuropeptide S (NPS) is an important neurotransmitter in the central nervous system with roles in the regulation of arousal, mood, food intake, and memory (18–28). Its only recognized receptor, NPS receptor 1 (NPSR1), is necessary for the tested biological effects of NPS (29). NPS was originally found to promote arousal and to inhibit all stages of sleep in rodents (18, 19). More recently, the *NPSR1-Y206H* mutation was identified as a causative mutation for the familial natural short sleep trait in humans (10), confirming that NPS–NPSR1 signaling is important for sleep regulation across different species.

NPS-expressing (NPS^+^) neurons have been found mainly in two pontine areas, the parabrachial nucleus (PB) and peri-locus coeruleus (peri-LC), in rodents (19, 30, 31). Recently, NPS^+^ neurons were identified in the central gray of the pons (CGPn) in mice (32). However, the functional role of these neurons in sleep regulation has not been fully elucidated. Here, we systematically investigated pontine NPS^+^ cells and their neural circuits to better understand the role of the NPS signaling system in sleep modulation. Our experiments revealed that NPS^+^ cells in the PB are wake-promoting. Interestingly, our results showed that peri-LC NPS^+^ cells play a minimal role in regulating sleep. Moreover, we found that NPS^+^ cells in the CGPn play a strong positive role in promoting sleep. Our experiments also provide evidence that the sleep/wake regulation activities of NPS^+^ neurons in the PB are partially regulated by NPS–NPSR1 signaling. With anterograde and retrograde tracing, we built the neuronal network of pontine NPS^+^ cells. Together, these results reveal that NPS and the neurons that express NPS have diverse roles in sleep regulation based on their location and that some of these neurons have antagonistic functions in sleep/wake promotion, depending on the context and network.

## Results

### PB NPS^+^ Neurons Are Wake-Promoting.

**Fig. 1.**
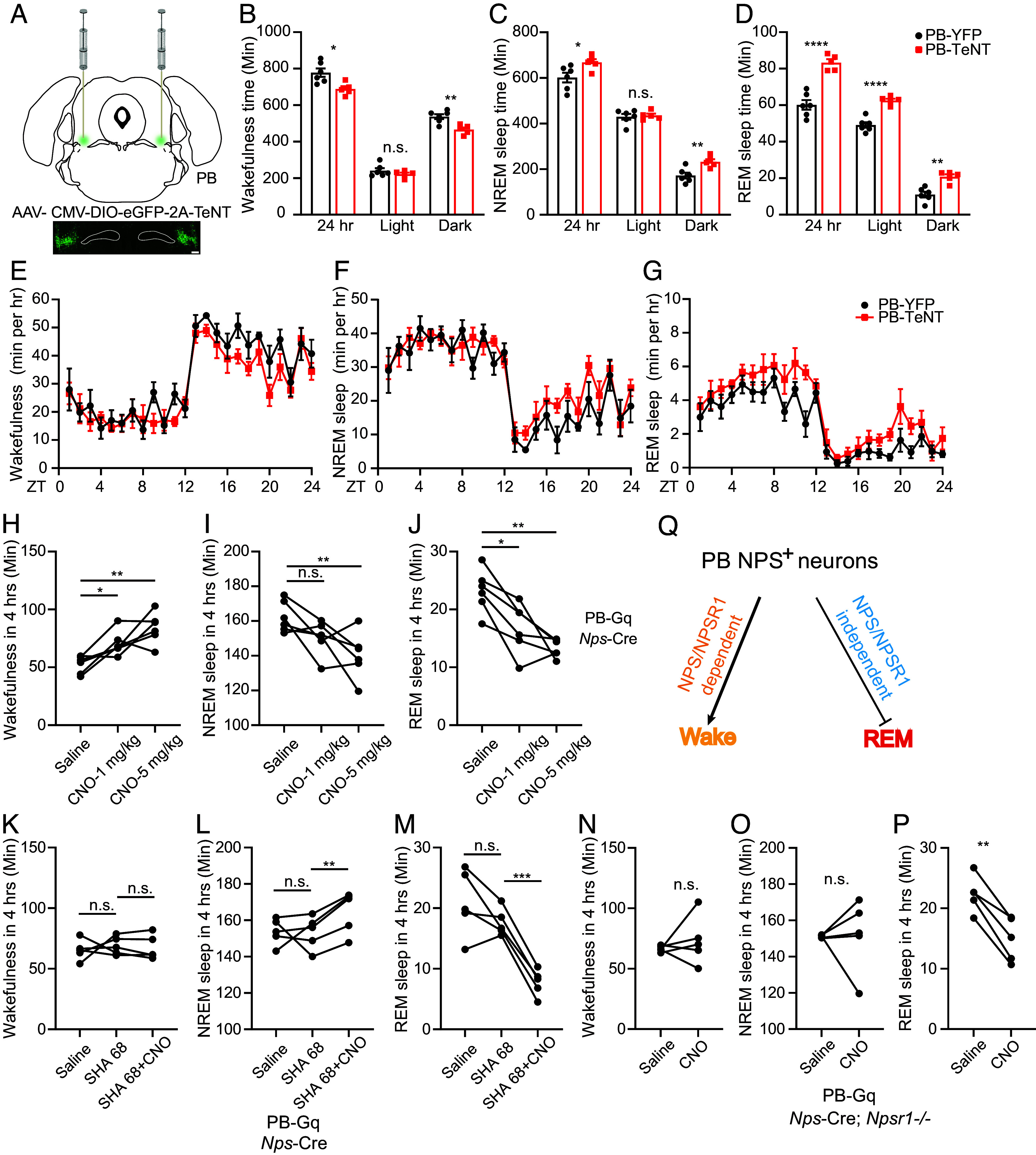
PB NPS^+^ neurons are wake-promoting. (*A*) Schematic of TeNT inhibition of PB NPS^+^ neurons. (*B*–*D*) Total wakefulness time (*B*), NREM sleep time (*C*), and REM sleep time (*D*) by EEG/EMG within 24 h, light phase, and dark phase were calculated in YFP (n = 6) and TeNT (n = 5) injected mice. (*E*–*G*) Total wakefulness (*E*), NREM sleep (*F*), and REM sleep (*G*) were plotted hourly for 24 h in YFP (n = 6) and TeNT (n = 5) injected mice. (*H*–*J*) Quantified results for total wakefulness (*H*), NREM (*I*), and REM (*J*) sleep recorded 4 h after saline or CNO injection at ZT6 in the hM3Dq (n = 6) injected mice. (*K*–*M*) Quantified results for total wakefulness (*K*), NREM (*L*), and REM (*M*) sleep recorded 4 h after saline (*Left* column), SHA 68 (*Middle* column) or SHA 68+CNO (5 mg/kg) (*Right* column) injection at ZT6 in the hM3Dq (n = 5) injected mice. (*N*–*P*) Quantified results for total wakefulness (*N*), NREM (*O*), and REM (*P*) sleep recorded 4 h after saline or CNO (5 mg/kg) injection at ZT6 in the hM3Dq (n = 5) injected *Nps*-Cre; *Npsr1*−/− mice. (*Q*) Schematic showing effect of NPS/NPSR1 signaling in the sleep/wake regulation activity of PB NPS^+^ neurons. *P < 0.05, **P < 0.01, ***P < 0.001, ****P < 0.0001, n.s. = not significant. Two-tailed Student’s *t* test for (*B*–*D*) and (*H*–*P*). Two-way ANOVA, post hoc Sidak’s multiple comparisons test for (*E*–*G*). RM one-way ANOVA, post hoc Dunnett's multiple comparisons test for (*H*–*M*). Error bars represent ± SEM. (Scale bar, 200 μm.)

To investigate the functions of NPS^+^ neurons, we generated a knock-in *Nps*-ires-Cre mouse line (*Nps*-Cre hereafter) (*SI Appendix*, Fig. S1*A*). After breeding with a Cre-dependent enhanced green fluorescent protein (EGFP) reporter line (EGFP-L10a) (*SI Appendix*, Fig. S1*B*) (33), GFP^+^ cells were observed in the PB, peri-LC, CGPn, and anterior hypothalamus (*SI Appendix*, Fig. S1 *C–E*). Scattered GFP^+^ cells were also seen in the lateral habenular nucleus (LHb), central medial nucleus of thalamus (CM), and amygdala (*SI Appendix*, Fig. S1 *F* and *G*). The expression pattern was highly consistent with that reported by Dake et al. (32). Consistent with previous studies, abundant *Nps* expression was observed in the PB and peri-LC (*SI Appendix*, Fig. S2 *C* and *D*) (30). We also observed relatively low *Nps* expression in the CGPn and anterior hypothalamus (*SI Appendix*, Fig. S2 *A* and *B*) (32). Because the PB contains one of the major populations of NPS^+^ neurons in rodents (19, 30, 31), we set out to investigate the function of these cells in sleep regulation. We first employed a loss-of-function strategy to inactivate these neurons by stereotaxic injection of an adeno-associated virus (AAV) vector expressing Cre-dependent tetanus toxin (TeNT) light chain (AAV-DJ/CMV-DIO-eGFP-2A-TeNT) into the *Nps*-Cre mice (Fig. 1*A*) (34, 35). Silencing of NPS^+^ neurons in the PB by TeNT increased sleep duration by about 90 min within 24 h (Fig. 1 *B*–*D*). The increased non-rapid-eye-movement (NREM) sleep was mainly seen during the dark phase (Fig. 1 *C* and *F*), while increased rapid-eye-movement (REM) sleep was observed in both light and dark phases (Fig. 1 *D* and *G*). Further electroencephalograph (EEG) data analysis revealed that increased NREM and REM bouts, but not their averaged bout length, were responsible for the overall increase in NREM and REM sleep time (*SI Appendix*, Fig. S3 *B*, *C*, *E*, and *F*). Silencing of PB NPS^+^ neurons led to fragmented wakefulness mainly in dark phase with increased wake bouts and reduced wake bout length (*SI Appendix*, Fig. S3 *A* and *D*). Moreover, a general increase in transitions between all sleep/wake states (*SI Appendix*, Fig. S3 *G–J*) was observed. Notably, an examination of relative EEG power spectra only showed an increase in wake δ power (1 to 4 Hz frequency) and a decrease in NREM δ power, with no influence on the REM power spectrum (*SI Appendix*, Fig. S4, *Left*).

Next, we used a chemogenetic approach (36) to activate PB NPS^+^ neurons. We injected AAV vectors encoding a modified excitatory muscarinic GPCR [AAV8/hSyn-DIO-hM3D(Gq)-mCherry] or the control mCherry (AAV8/hSyn-DIO-mCherry) virus into the PB of the *Nps*-Cre mice (*SI Appendix*, Fig. S5*A*). Once neurons express the Gq receptor, they can be activated by systemic clozapine-N-oxide (CNO) administration (36). By whole-cell patch-clamp recording of neuronal activities in acute brain slices, we showed that bath application of CNO depolarized the membrane potential and increased the firing rates of mCherry+ neurons, thus confirming the excitatory effect of CNO on hM3D(Gq)-expressing NPS^+^ neurons (*SI Appendix,* Fig. S5*B*). Two doses of CNO (1 mg/kg and 5 mg/kg; intraperitoneal injection) were administered for each group during the light phase to track the wake-promoting effect. Consistent with the TeNT silencing results, activation of PB NPS^+^ cells using the chemogenetic approach significantly increased wakefulness at the cost of both NREM and REM sleep (Fig. 1 *H*–*J*), while CNO injection did not cause significant changes in sleep/wake state in the mCherry-infected mice (*SI Appendix,* Fig. S5 *C–E*). These data suggest that PB NPS^+^ neurons are primarily wake-promoting.

### NPSR1 Inhibition Partially Blocks the Effects of PB NPS^+^ Neurons on Sleep Regulation.

NPSR1 is the only receptor for NPS identified to date (29). To evaluate the role of NPS–NPSR1 signaling in the sleep/wake-promoting effects of PB NPS^+^ cells, we performed a chemogenetic activation (Gq) experiment while blocking NPSR1 using pharmaceutical and genetic strategies. We repeated the activation (Gq) experiment in the presence of SHA 68, a potent antagonist of NPSR1 in vivo (37, 38). As shown in Fig. 1 *K*–*M*, SHA 68 blocked the wake-promoting effect of the CNO but did not inhibit the activity of the CNO in reducing REM sleep. Similar results were observed in the *Nps*-Cre; *Npsr1*−/− mice (Fig. 1 *N*–*P*). These results imply that NPS–NPSR1 signaling in the PB is required for the wake-promoting but not the REM sleep-inhibiting effects of PB NPS^+^ neurons (Fig. 1*Q*).

### Peri-LC NPS^+^ Neurons Have Minimal Effect on Sleep.

**Fig. 2.**
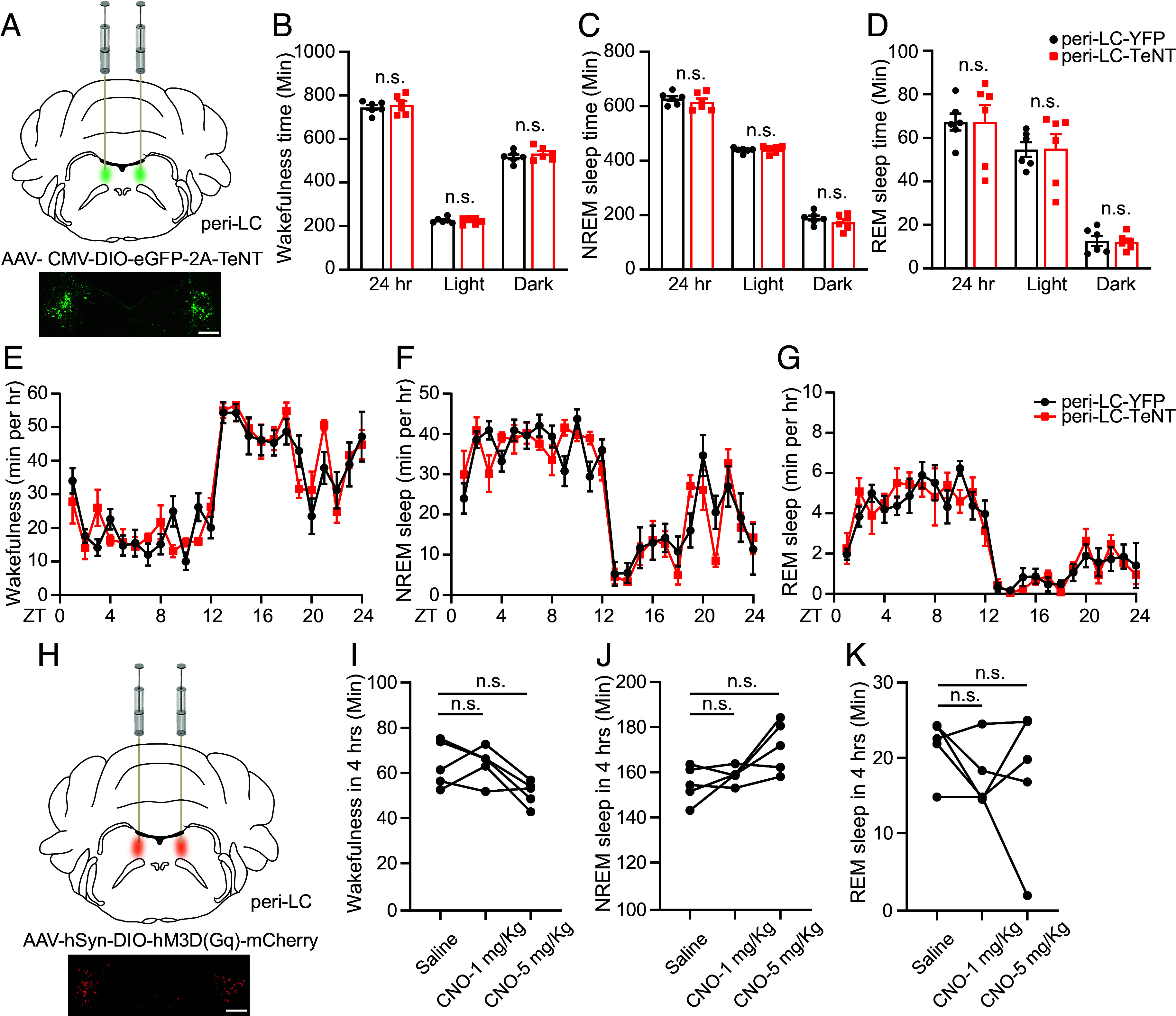
Peri-LC NPS^+^ neurons have minimal role in regulating sleep. (*A*) Schematic of TeNT inhibition of peri-LC NPS^+^ neurons. (*B*–*D*) Total wakefulness time (*B*), NREM sleep time (*C*), and REM sleep time (*D*) by EEG/EMG within 24 h, light phase and dark phase were calculated in YFP (n = 6) and TeNT (n = 6) injected mice. (*E*–*G*) Total wakefulness (*E*), NREM sleep (*F*), and REM sleep (*G*) were plotted hourly for 24 h in YFP (n = 6) and TeNT (n = 6) injected mice. (*H*) Schematic slice of chemogenetic activation of peri-LC NPS^+^ neurons. (*I*–*K*) Quantified results for total wakefulness (*I*), NREM (*J*), and REM (*K*) sleep recorded 4 h after saline or CNO injection at ZT6 in the hM3Dq (n = 5) injected mice. n.s. = not significant. Two-tailed Student’s *t* test for (*B*–*D*). Two-way ANOVA, post hoc Sidak’s multiple comparisons test for (*E*–*G*). RM one-way ANOVA, post hoc Dunnett's multiple comparisons test for (*I*–*K*). Error bars represent ± SEM. (Scale bar, 200 μm.)

We next investigated the role of NPS^+^ neurons in the peri-LC (the other major population) in sleep regulation. Silencing of peri-LC NPS^+^ neurons (Fig. 2*A*) produced no significant changes in various sleep parameters, including sleep duration, wake/sleep transitions, bouts, and averaged bout length (Fig. 2 *B*–*G* and *SI Appendix*, Fig. S6). The exceptions to this were alterations in wake and REM δ power (*SI Appendix*, Fig. S7, *Left*). Chemogenetic activation of peri-LC NPS^+^ cells had no significant effect on sleep parameters (Fig. 2 *H*–*K*). Collectively, these findings suggest a less important role for peri-LC NPS^+^ neurons in modulating sleep.

### PB and Peri-LC NPS*^+^* Neurons Are Wake- and REM-Active.

**Fig. 3.**
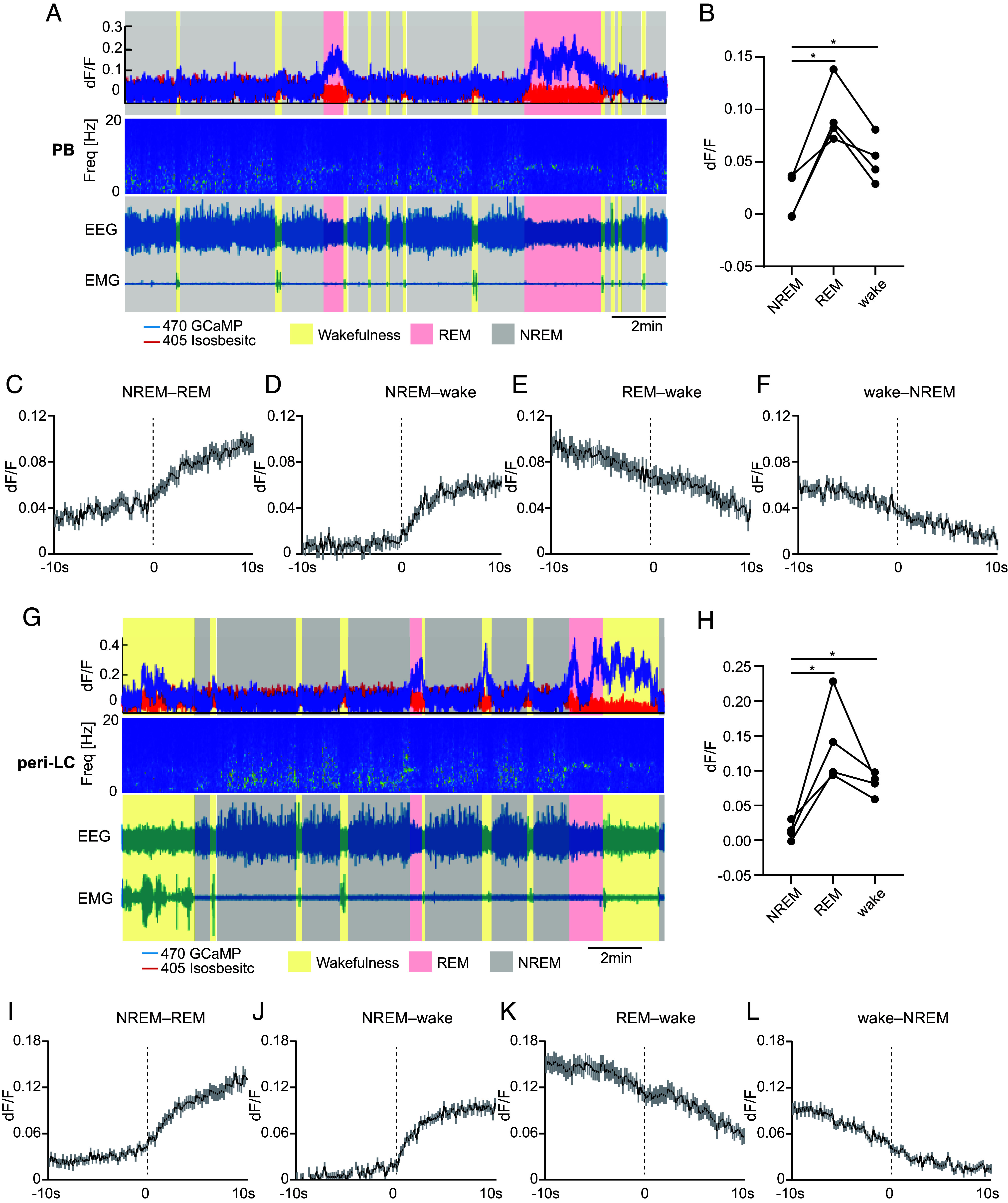
PB and peri-LC NPS^+^ neurons are wake- and REM-active. (*A*) Representative EEG power spectrogram, EEG, EMG, and fluorescence traces across spontaneous sleep-wake states of PB fiber photometry recording. (*B*) Quantified fluorescence signal across different sleep/wake states from n = 4 mice of PB recording. (*C*–*F*) Average fluorescence signals aligned to NREM–REM (*C*, 60 transitions), NREM–wake (*D*, 60 transitions), REM–wake (*E*, 60 transitions), and wake–NREM state transitions (*F*, 60 transitions) (n = 4 mice) for PB recording. (*G*) Representative EEG power spectrogram, EEG, EMG, and fluorescence traces across spontaneous sleep-wake states of peri-LC fiber photometry recording. (*H*) Quantified fluorescence signal across different sleep/wake states from n = 4 mice of peri-LC recording. (*I*–*L*) Average fluorescence signals aligned to NREM–REM (*I*, 60 transitions), NREM–wake (*J*, 60 transitions), REM–wake (*K*, 60 transitions), and wake–NREM state transitions (*L*, 60 transitions) (n = 4 mice) for peri-LC recording. *P < 0.05. RM one-way ANOVA, post hoc Dunnett's multiple comparisons test (*B* and *H*). Shaded areas represent ± SEM in (*C*–*F* and *I*–*L*).

To study NPS^+^ neuronal activity across physiological wake/sleep states, we used fiber photometry with EEG/electromyograph (EMG) recording. A Cre-dependent AAV vector encoding the fluorescent calcium indicator GCaMP6s (AAV1/Syn-Flex-GCaMP6s-WPRE-SV40) was injected into the PB or peri-LC of the *Nps*-Cre mice. We implanted both a fiber optic probe and EEG/EMG electrodes for simultaneous sleep/wake recordings (*SI Appendix*, Fig. S8 *A* and *C*). We found that the NPS^+^ neurons in the PB are REM- and wake-active and NREM-inactive (Fig. 3 *A*–*F* and *SI Appendix*, Fig. S8*B*). Interestingly, the peri-LC NPS^+^ neurons were also wake- and REM-active (Fig. 3 *G*–*L* and *SI Appendix*, Fig. S8*D*) indicating that while peri-LC NPS^+^ neurons are not required for sleep regulation, their activity is coupled to the sleep/wake state. Like many other wake- and REM-active neurons, the signals for both groups of neurons quickly dropped to baseline after each wake–NREM transition. Notably, the fluctuations of the signals during wake or REM states suggest that the activity of these NPS^+^ neurons may be correlated with other behavioral states.

### Silencing NPS^+^ Neurons or Knockdown NPS in CGPn Increases Wakefulness.

**Fig. 4.**
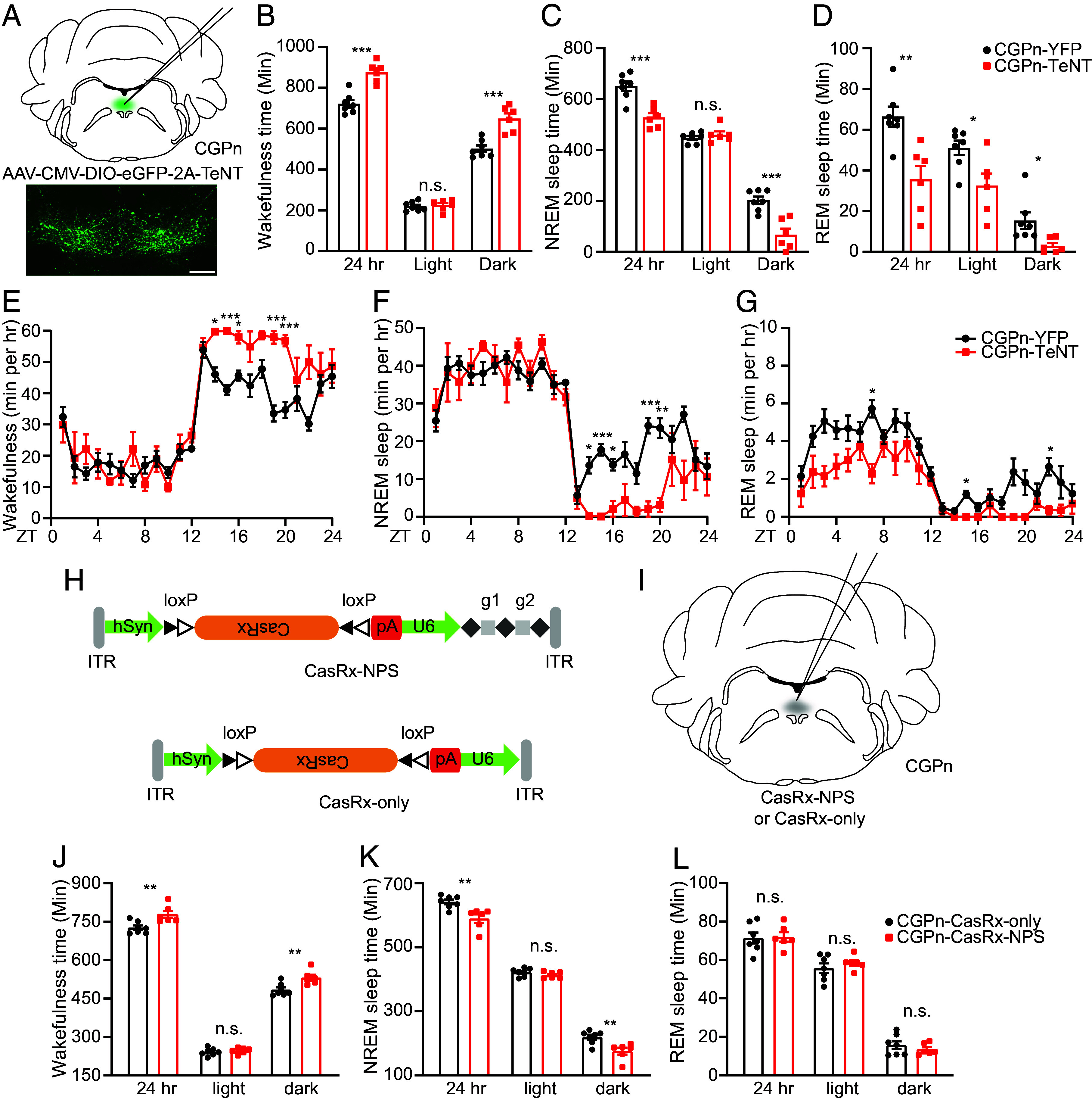
CGPn NPS*^+^*neurons are sleep-promoting. (*A*) Schematic of TeNT inhibition of CGPn NPS^+^ neurons. (*B*–*D*) Total wakefulness time (*B*), NREM sleep time (*C*), and REM sleep time (*D*) by EEG/EMG within 24 h, light phase, and dark phase were calculated in YFP (n = 7) and TeNT (n = 6) injected mice. (*E*–*G*) Total wakefulness (*E*), NREM sleep (*F*), and REM sleep (*G*) were plotted hourly for 24 h in YFP (n = 7) and TeNT (n = 6) injected mice. (*H*) AAV design carrying Cre-dependent CasRx with (*Upper*, CasRx-NPS) or without (*Lower*, CasRx-only) gRNAs targeting indicated mRNA between the AAV inverted terminal repeats (ITRs). (*I*) Schematic of CasRx virus delivery to CGPn NPS^+^ neurons. (*J*–*L*) Total wakefulness time (*J*), NREM sleep time (*K*), and REM sleep time (*L*) by EEG/EMG within 24 h, light phase and dark phase were calculated in CasRx-only (n = 7) and CasRx-NPS (n = 6) injected mice. *P < 0.05, **P < 0.01, ***P < 0.001, n.s. = not significant. Two-tailed Student’s *t* test for (*B*–*D* and *J*–*L*). Two-way ANOVA, post hoc Sidak's multiple comparisons test for (*E*–*G*). Error bars represent ± SEM. (Scale bar, 200 μm.)

CGPn NPS^+^ neurons were previously marked as an “uncharacterized population” in the human brain (39). Although *Nps* expression is lower in the CGPn than in the PB and peri-LC (*SI Appendix*, Fig. S2), we tested the participation of CGPn NPS^+^ neurons in sleep regulation. Interestingly, TeNT inactivation of CGPn neurons dramatically decreased the sleep duration (~150 min in 24 h) compared to that in control mice injected with virus encoding only yellow fluorescent protein (YFP) (Fig. 4 *A*–*G*). NREM sleep changes were only observed during the dark phase (Fig. 4 *C* and *F*), while changes in REM sleep were observed in both the light and dark phases (Fig. 4 *D* and *G*). The reduced NREM and REM sleep time was mainly attributed to decreased NREM and REM bout numbers, which was accompanied with a significantly decreased wake bout number and markedly increased wake bout length (*SI Appendix*, Fig. S9 *A–F*). These observations, along with the overall decreases in all wake/sleep transitions (*SI Appendix*, Fig. S9 *G–J*), suggest that silencing of CGPn NPS^+^ neurons may promote wakefulness by sustaining wakefulness. Reduced NREM sleep during the dark phase usually is accompanied by increased δ power in the subsequent light phase. Consistently, we observed an enhanced δ power during the early light phase in CGPn TeNT-injected mice (*SI Appendix*, Fig. S10*A*). Relative spectral analysis revealed significant alterations in both the δ and θ (6 to 9 Hz frequency) powers for all three sleep/wake states, especially during the light phase (*SI Appendix*, Fig. S10 *B–D*, *Left*).

To confirm the role of NPS in these CGPn neurons, we developed a virus to specifically knock down NPS (AAV8/Syn-DIO-CasRx-U6-NPS gRNA, CasRx-NPS) (Fig. 4*H*). We confirmed that this virus efficiently down-regulated *Nps* mRNA in the PB (*SI Appendix*, Fig. S11). Knockdown of *Nps* in CGPn neurons significantly decreased the sleep duration (~52 min in 24 h) mainly during the dark phase (Fig. 4 *J*–*L*). This change was in the same direction as that elicited by TeNT-mediated neuronal silencing (Fig. 4 *B*–*D*), albeit to a less extent. Together, these results indicate that NPS in the CGPn may play a role in promoting sleep.

### Activation of CGPn NPS^+^ Neurons Promotes Sleep.

**Fig. 5.**
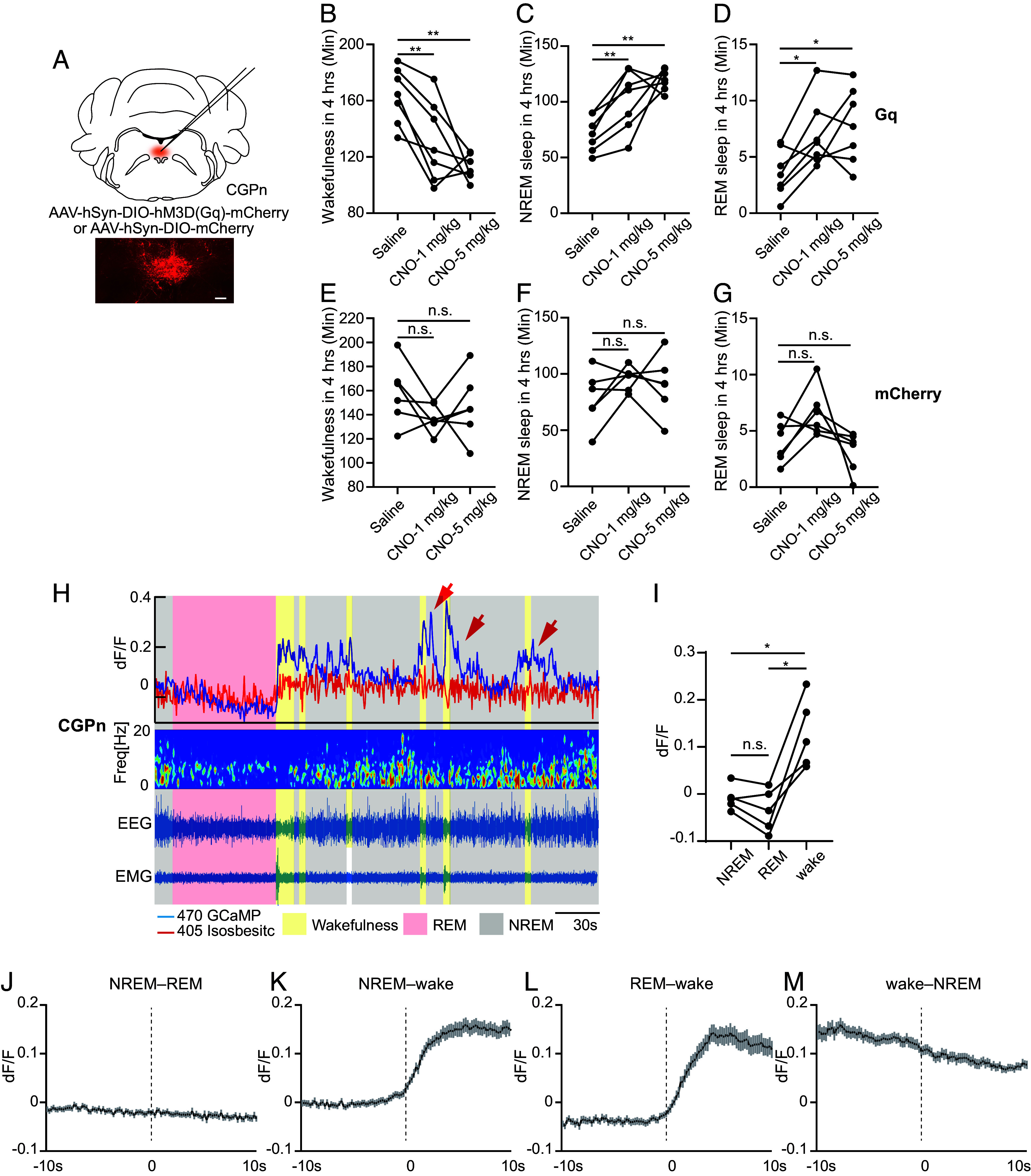
CGPn NPS^+^ neurons are wake-active. (*A*) Schematic of chemogenetic activation of CGPn NPS^+^ neurons. (*B*–*D*) Quantified results for total wakefulness (*B*), NREM (*C*), and REM (*D*) sleep recorded 4 h after saline or CNO injection at ZT14 in the hM3Dq (n = 7) injected mice. (*E*–*G*) Quantified results for total wakefulness (*E*), NREM (*F*), and REM (*G*) sleep recorded 4 h after saline or CNO injection at ZT14 in the mCherry (n = 6) injected mice. (*H*) Representative EEG power spectrogram, EMG, and fluorescence traces across spontaneous sleep/wake states. Red arrows indicate substantial GCaMP signal spikes after short wakefulness. (*I*) Quantified fluorescence signals across different sleep/wake states from n = 5 mice. (*J*–*M*) Average fluorescence signal aligned to NREM–REM (*J*, 75 transitions), NREM–wake (*K*, 75 transitions), REM–wake (*L*, 66 transitions), and wake–NREM state transitions (*M*, 75 transitions) (n = 5 mice). *P < 0.05, **P < 0.01, n.s.=not significant. RM one-way ANOVA, post hoc Dunnett’s multiple comparisons test for (*B*–*G*). RM one-way ANOVA, post hoc Tukey’s multiple comparisons test for (*I*). Shaded areas in (*J*–*M*) represent ± SEM.

We next used the chemogenetic approach to determine the effect of activating CGPn NPS^+^ neurons on sleep/wake behavior. CNO was administered at ZT14 when mice are mostly awake to track the sleep-promoting effect. Activation of CGPn NPS^+^ cells significantly increased both NREM and REM sleep after CNO administration (Fig. 5 *A*–*D*), whereas CNO injection did not cause significant changes in sleep/wake states in the mCherry-infected mice (Fig. 5 *E*–*G*), providing evidence that CGPn NPS^+^ cells are primarily sleep-promoting.

### Activity of CGPn NPS^+^ Neurons Is Coupled with Vigilance States.

With fiber photometry and EEG/EMG simultaneous recording (*SI Appendix*, Fig. S12*A*), we found that the activity of CGPn NPS^+^ neurons is coupled with sleep/wake states, as these neurons are significantly active during wakefulness, maintain a background level of activity during NREM sleep, and show a trend toward even lower activity during REM sleep, although not significant (Fig. 5 *H*–*M* and *SI Appendix*, Fig. S12*B*). Intriguingly, these neurons are sensitive to wake episodes; even a short period of wakefulness (less than 5 s) was sufficient to evoke a substantial spike in GCaMP signal (Fig. 5*H*, red arrow). This is consistent with the possibility that activated NPS^+^ neurons in this region during a wake episode play a role in assisting the wake/NREM transition (*SI Appendix*, Fig. S*9*). Examination of the signals revealed that the activity not only is associated with wakefulness but also persists at a low level after the wake-to-NREM transition before dropping to background (Fig. 5 *H* and *M*).

## Pontine NPS^+^ Neurons Connect with Other Brain Regions.

**Fig. 6.**
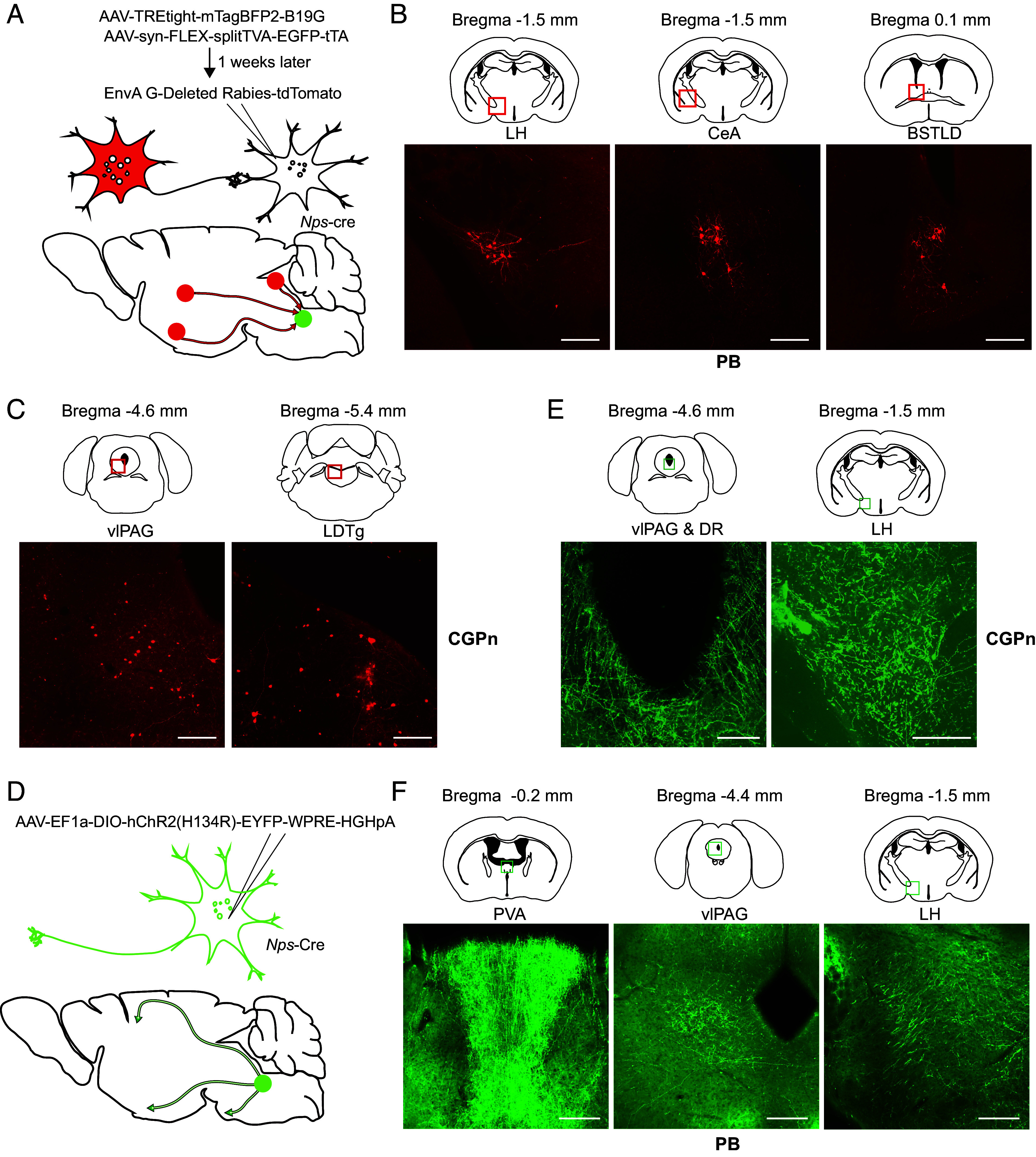
Retrograde and anterograde tracing of NPS^+^ cells. (*A*) Schematic strategy for identifying upstream neurons using RV-mediated retrograde tracing. (*B*) RV-labeled presynaptic neurons (red squares) of PB NPS^+^ cells. (*C*) RV-labeled presynaptic neurons (red squares) of CGPn NPS^+^ cells. (*D*) Schematic strategy for tracing projections of target neurons using virus [AAV5/EF1a-DIO-hChR2 (H134R)-EYFP]. (*E*) Expression of eYFP in PB NPS^+^ neuron terminals at indicated regions (green squares). (*F*) Expression of eYFP in CGPn NPS^+^ neuron terminals at indicated regions (green squares). Representative brain sections from n = 3 mice. (Scale bar, 200 μm.) BSTLD, laterodorsal bed nucleus of the stria terminalis; CeA, central amygdala; DMH, dorsomedial hypothalamus; DR, dorsal raphe; LDTg, laterodorsal tegmental nucleus; LH, lateral hypothalamus; PVA, anterior nucleus of paraventricular thalamus; PVT, paraventricular thalamic nucleus; vlPAG, ventrolateral periaqueductal gray.

To establish the NPS^+^ neuronal circuitry, we inspected direct inputs from upstream neurons by performing Cre-dependent rabies virus (RV)-based monosynaptic tracing from NPS^+^ neurons. An avian retroviral receptor (TVA) fused with EGFP and a rabies glycoprotein (B19G) were expressed in neuronal populations by injecting two Cre-inducible AAV vectors (AAV1/syn-FLEX-splitTVA-EGFP-tTA and AAV1/TREtight-mTagBFP2-B19G) into the PB, peri-LC, and CGPn of *Nps*-Cre mice. A modified RV expressing tdTomato (RVdG-tdTomato+EnvA) was injected 1 wk later to infect the TVA-expressing target neurons (Fig. 6*A*) and label their presynaptic inputs (40–42). Inputs to the PB and peri-LC were widely distributed. The tdTomato-labeled input neurons of the PB were found with substantial fractions in the lateral hypothalamus (LH), the central amygdala (CeA), and the laterodorsal bed nucleus of the stria terminalis (BSTLD) (Fig. 6*B*). Beyond these three nuclei, the lateral preoptic area (LPO) and ventromedial hypothalamus were also found to be direct upstream nuclei of the peri-LC (*SI Appendix*, Fig. S13*A*). For CGPn NPS^+^ neurons, the tdTomato-labeled input neurons were found within the laterodorsal tegmental nucleus (LDTg) and the ventrolateral periaqueductal gray (vlPAG) (Fig. 6*C*). Thus, while CGPn NPS^+^ neurons mainly receive local innervation, the PB and peri-LC NPS^+^ neurons integrate a much wider range of inputs from more distal regions in the midbrain.

To examine the relevant efferent projections of the NPS^+^ neurons, we injected the AAV vector carrying Cre-dependent ChR2-EYFP transgenes [rAAV5/EF1a-DIO-hChR2(H134R)-EYFP] into the PB, peri-LC, and CGPn separately (Fig. 6*D*). As shown in Fig. 6*E*, CGPn NPS^+^ neurons projected primarily to the LH, vlPAG, and dorsal rhaphe (DR), whereas PB NPS^+^ neurons projected more diversely to several regions (Fig. 6*F* and *SI Appendix*, Fig. S13*B*). In addition to the LH and vlPAG, which are known sleep centers, PB NPS^+^ neurons projected to areas including the anterior nucleus of paraventricular thalamus (PVA), the paraventricular thalamic nucleus (PVT), the LPO, the dorsomedial hypothalamus (DMH), and the median preoptic nucleus (Fig. 6*F* and *SI Appendix*, Fig. S13*B*). In addition to the PVA, vlPAG, LH, and LPO, peri-LC NPS^+^ neurons also projected to the pedunculopontine tegmental nucleus (*SI Appendix*, Fig. S13*C*). Therefore, the LH and vlPAG are the common regions receiving projections from the PB, peri-LC, and CGPn regions. The connections revealed here provide the circuit basis for direct coordination of the PB and CGPn NPS^+^ neurons with sleep/wake neurons in the brain (*SI Appendix*, Fig. S13*D*). Comparison of the upstream and downstream nuclei of each region can provide insight regarding the different sleep/wake regulatory functions of PB, peri-LC, and CGPn NPS^+^ neurons.

## Discussion

NPS is one of the least abundant neuropeptides in the rodent and human brains both in terms of levels and number of expressing neurons (31, 39). Direct probing of endogenous *Nps* mRNA or protein in rat and mouse showed that NPS is mainly expressed by a few well-defined pontine neuronal clusters: the peri-LC and PB (previously reported as Kölliker-Fuse nucleus) (19, 30, 31). Here, using a *Nps*-Cre driven GFP reporter, we were able to identify several NPS^+^ nuclei, including the CGPn. Our *Nps*-Cre strategy enabled the GFP reporter to be expressed by the strong CAG promoter, and the observed expression pattern is nearly identical to that generated by another recently reported *Nps*-Cre line (32).

Human NPS^+^ cells were identified in pons mainly in the CGPn, PB, and peri-LC (39). The CGPn cluster in humans contains ~11% of the NPS^+^ cells in the brainstem (39). The corresponding “peri-ventricular cluster” in rats was identified as NPS^+^ neurons with unknown function (39). Interestingly, the locations of CGPn NPS^+^ clusters vary among species. Human and rat CGPn NPS^+^ neurons are dorsal to the posterodorsal tegmental nucleus (PDTg) and closely adjacent to the fourth ventricle (39). However, in mice, we found that NPS^+^ neurons are ventral to the PDTg and not adjacent to the fourth ventricle (*SI Appendix*, Fig. S1*C*). The PB cluster contains the vast majority (84%) of NPS^+^ cells in the human brainstem (39), and it contributes the majority (71%) of NPS^+^ cells in mice (31). The peri-LC cluster only contains a few scattered neurons (4 to 5% of all NPS^+^ cells) in humans (39), although it is another prominent NPS^+^ cell source in rodents (19, 31).

Central administration of NPS in rodents promotes arousal (19). Thus, NPS has been considered a wake-promoting neuropeptide (18). Consistent with this, we observed that NPS–NPSR1 signaling in the PB (which contains the largest population of NPS^+^ cells in mice) plays a role in promoting wakefulness. However, peri-LC NPS^+^ neurons do not seem to play major role despite the known role of the LC in sleep regulation. We further tested the NPS^+^ neurons in the area with the next tier of expression, the CGPn, for their possible participation in sleep regulation. Intriguingly, we discovered that CGPn NPS^+^ neurons act as a repressor of wakefulness (Fig. 4), and NPS in these neurons functions as a somnogen (Fig. 4 *H*–*L*), revealing that NPS can be either wake- or sleep-promoting. Also supporting the diverse roles of NPS is the finding that mice lacking *Nps* did not show an altered sleep phenotype (*SI Appendix*, Fig. S14). Another interesting observation here is that silencing NPS^+^ neurons in the PB caused a general increase in all sleep/wake state transitions, whereas silencing them in the CGPn led to a general decrease in all state transitions. More study is needed to gain insight into this finding. Nonetheless, the observation that silencing NPS^+^ neurons in the CGPn greatly increased wakefulness, at least partly due to a dramatic increase in wake bout length and decrease in wake bout number, suggests the possibility that CGPn NPS^+^ neurons may promote sleep by inhibiting wakefulness.

Like many sleep/wake neurons, the PB and peri-LC NPS^+^ neurons are both wake- and REM-active (Fig. 3). Contrary to previously reported sleep-promoting neurons, which are mostly NREM active (43, 44), CGPn NPS^+^ neurons are only wake-active with an intriguingly sustained low activity in the following NREM sleep (Fig. 5*H*). Although we know very little about the functional role of wake-active-only neurons, some REM-active hypothalamic neurons have been shown to have specific functions in memory formation and forgetting processes (45, 46). Further studies are needed to better define the functional role of these CGPn NPS^+^ neurons in modulating the finer processing of sleep/wake transition and/or maintenance.

We noticed that some brain regions, such as the LH and vlPAG, receive projections from both the sleep-promoting CGPn and wake-promoting PB NPS^+^ neurons (Fig. 6 and *SI Appendix*, Fig. S13). Determining how the downstream neurons integrate these different signals to modulate sleep behavior of the whole organism will require further investigation. Considering that both humans and mice with the *Npsr1*-Y206H mutation exhibit a short sleep phenotype and that neurons from *Npsr1-*Y206H mice are hypersensitive to NPS (10), we speculate that this mutation may change the counterbalance of signals from different NPS^+^ cells, thus altering sleep duration. For example, if a sleep homeostatic center receives more inputs from wake-promoting than sleep-promoting neurons, there will be higher wake-promoting signals at this center with mutant receptor than at those with wild-type receptor due to the heightened sensitivity of the mutant receptor. This will produce an altered homeostatic state that favors wakefulness. Efferent projection tracing showed that PB NPS^+^ neurons project to more regions than CGPn NPS^+^ neurons. Another possibility is that increased receptor activity in these wake-promoting regions may shift the balance toward wakefulness (short sleep). Further investigation of the mechanism is necessary to distinguish these possibilities.

In addition to arousal/sleep, NPS is involved in the regulation of food intake, mood, and fear-related memory in mice (21–27). It is conceivable that pontine NPS^+^ neurons produce the vast majority of NPS in the brain based on the observed expression levels, and this NPS can be transported from the pontine area to other regions via long-range projections to regulate the corresponding physiology. However, we also observed GFP^+^ cells in the anterior hypothalamus, LHb, and amygdala (*SI Appendix*, Fig. S1 *E–G*), which are the brain areas responsible for social reward, food intake, mood, fear-related memory, etc. Whether local NPS signaling plays a role in regulating these physiological processes will be an interesting focus for future studies.

Sleep/wake behavior modulation is a choreography that involves intricate and complex systems working synergistically together (47). Here, we focus on one small component, NPS signaling. NPS and NPSR1 have been thought to have a one-on-one functional role. Intriguingly, we found that both SHA 68 treatment and *Npsr1* knockout in mice mostly abolished the wake-promoting effect of NPS in the PB but not the effect on REM reduction, suggesting that the role of PB NPS in REM regulation involves additional layers of complexity which warrants further investigation. We also observed that silencing NPS neurons in both PB and CGPn showed an effect on NREM mainly in the dark phase whereas the effect on REM is found in both light and dark phases. One possible explanation for this difference is that different projecting areas of the NPS neurons may participate in different sleep state modulation. Interestingly, some of the regions known to regulate REM (e.g., LH, vlPAG) (48) do receive projections from NPS neurons. Moreover, finer fluctuations and rhythms in calcium activity recordings in various sleep/wake states were observed and may indicate a correlation of NPS neuron activity to other behaviors (food intake, mood, etc.). Adding to this complexity is the possibility of multiple co-expressing factors in various neuronal nuclei working coordinately to modulate these behaviors. Further in-depth studies are needed to shed light on how different sleep/wake states are fine-tuned to establish the whole organism’s sleep/wake behavior and how neurons at the same and different areas coordinate with each other to modulate multiple behaviors for the entire organism.

## Materials and Methods

Detailed descriptions of animal studies; EEG/EMG implantation, recording and scoring; fiber photometry; electrophysiological recording; anterograde and retrograde tracing; histology and immunohistochemistry; fluorescence imaging; and CasRx AAV vector can be found in **SI Appendix*, SI Materials and Methods*.

### Animal Resources.

*Nps*-Cre mice were generated by CRISPR/Cas9. The IRES-Cre DNA cassette was inserted after the TGA stop codon of the *Nps* open reading frame. Briefly, IRES-Cre was constructed in the pEGFP-N1 backbone with ~3-kb homologous sequences on both 5′ and 3′ sides. As previously described (9–11), plasmid was purified (MEGAclear kit, Life Technologies) in RNase-free water. Super-ovulated female FVB/N mice were mated to FVB/N males, and fertilized zygotes were collected from oviducts. Cas9 protein (50 ng/mL), sgRNA (20 ng/mL), and IRES-Cre DNA plasmid (20 ng/mL) were mixed and injected into the pronucleus of fertilized zygotes. Injected zygotes were implanted into oviducts of pseudo-pregnant CD1 female mice. Founders were genotyped by PCR and sequencing. Out of 22 pups genotyped, 3 were positive for the knock-in. Mice were then backcrossed onto a C57BL/6J (The Jackson Laboratory, RRID: IMSR_JAX:000664) background for at least five generations before phenotyping to dilute potential off-target effects. Two independent lines were chosen for experimentation, which gave similar results in all the tests. *Nps* knock-out mice were obtained as a by-product when generating the knock-in mice. Two founders were found to be missing the whole coding region. *Nprs1* knock-out mice were generated as previously described (10).

B6;129S4-Gt(ROSA)26Sor^tm9(EGFP/Rpl10a)Amc/J^ (EGFP-L10a, The Jackson Laboratory, RRID: IMSR_JAX:024750024750) mice were originated from The Jackson Laboratory.

### Neuron Silencing with Tetanus Toxin.

For chronic neural perturbation experiments, *Nps*-Cre mice were infused with AAV-DJ-CMV-DIO-eGFP-2A-TeNT virus (Stanford Viral Core) (150 to 300 nL) bilaterally into the peri-LC (bregma: AP: −5.6 mm, ML: ±0.7 mm, DV: −3.7 mm), PB (bregma: AP: −4.9 mm, ML: ±1.7 mm, DV: −3.8 mm), and CGPn (bregma: AP: −5.6 mm, ML: 0.0 mm, DV: −4.5 mm). For the control experiment, AAV-EF1a-DIO-eYFP virus (UNC Vector Core) was infused into each targeted area. EEG/EMG implantation was carried out after virus infusion. Behavioral experiments were conducted 3 wk later to allow for sufficient recovery and virus expression. Each mouse was recorded for two continuous days, and the data were averaged across 2 d.

### Chemogenetic Manipulation.

*Nps*-Cre mice were infused with rAAV8/hSyn-DIO-hM3D(Gq)-mCherry (Addgene, Cat # 44361-AAV8, RRID: Addgene_ 44361) into the PB, peri-LC, and CGPn areas using the above coordinates and fitted with EEG/EMG headsets as described above. Control mice were infused with rAAV8/hSyn-DIO-mCherry (Addgene, Cat # 50459-AAV8, RRID: Addgene_ 50459) virus and fitted with the same headsets. Five days before data acquisition, mice received daily administration of saline to make them fully acclimated to intraperitoneal injection.

## Supplementary Material

Appendix 01 (PDF)

## Data Availability

All study data are included in the article and/or *SI Appendix*.
