# Supplementary material for: Diverse roles of pontine NPS-expressing neurons in sleep regulation
Source: Proc Natl Acad Sci U S A. 2024 Feb 21;121(9):e2320276121. doi: 10.1073/pnas.2320276121 (PMC10907243; doi:10.1073/pnas.2320276121)
Supplement: Supplementary file 1 — Appendix 01 (PDF) [file pnas.2320276121.sapp.pdf]

Supporting Information for
Diverse roles of pontine NPS-expressing neurons in sleep regulation

Lijuan Xing, Xianlin Zou, Chen Yin, John M. Webb, Guangsen Shi, Louis J. Ptáček, and Ying-Hui Fu

Corresponding authors: Ying-Hui Fu, Louis J. Ptáček
Email: Ying-Hui.Fu@ucsf.edu (Y-H.F.); ljp@ucsf.edu (L.J.P.)

This PDF file includes:

SI Materials and Methods
Figures S1 to S14
SI References

SI Materials and Methods

Animal studies. As described in previous studies (1-3), all experimental animals were singly housed in light/dark (LD) 12:12 and given *ad libitum* access to food and water. Male mice were used for all behavioral experiments. Mice were at least 8 weeks old at the time of surgery. For the studies of Designer Receptors Exclusively Activated by Designer Drugs (DREADDs)/fiber photometry, *Nps*-Cre mice were randomly assigned to experimental or control groups that receiving different virus injections. For the experiments that compared sleep time, the light intensity of the room was strictly controlled between 70–90 lux. All experimental protocols were approved by the University of California, San Francisco IACUC following NIH guidelines for the Care and Use of Laboratory Animals.

EEG/EMG implantation, recording and scoring. The EEG/EMG surgery, recroding and scoring were performed as described in previous studies (1-3). Animals were anesthetized with 2% isoflurane and placed in a stereotaxic head frame on a heating pad. Ophthalmic ointment was applied to the eyes to prevent drying. A midline incision was made down the scalp, and four guide holes were made using a 23-gauge surgical needle epidurally over the frontal cortical area (1 mm anterior to bregma, 1 mm lateral to the midline) and over the parietal area (3 mm posterior to bregma, 2.5 mm lateral to midline). One ground screw and three screws with leads were placed into the skull through the holes. The screws with leads were then soldered onto a 6-pin connector EEG/EMG headset (Pinnacle Technologies, Lawrence, KS). For EMG recordings, EMG leads from the headset were placed into the neck muscle. The headset was then covered with black dental cement to form a solid cap atop the mouse's head. The incision was then closed with VetBond (3M, Santa Cruz Biotechnology), and animals were given a subcutaneous injection of marcaine (0.05 mg/kg) prior to recovery on a heating pad. Behavioral experiments were conducted 3 weeks later to allow for sufficient recovery.

For EEG/EMG recordings, mice were singly housed and habituated to the recording cable for 7 days in LD 12:12. Tethered pre-amplifiers were attached to the headset of the mice. The signals were relayed through the commutators, which allowed the animal to move freely. Data were acquired through the Sirenia software package (Pinnacle Technologies) (4). All data were sampled at 400 Hz.

Sleep of TeNT-treated and control animals was automatically scored by Spindle in 4-s epochs. Other EEG/EMG recordings were scored semi-automatically by Sirenia Sleep Pro software in 10-s epochs for wakefulness, NREM sleep, and REM sleep, and then subsequently hand-scored by researchers blinded to genotype with the assistance of spectral analysis using the fast Fourier transform (FFT). In general, wakefulness was defined as desynchronized low-amplitude EEG and heightened tonic EMG activity with phasic bursts. NREM sleep was defined as synchronized, high-amplitude, low-frequency (0.5–4 Hz) EEG and substantially reduced EMG activity compared with wakefulness. REM sleep was defined as having a pronounced theta rhythm (4–9 Hz) with no EMG activity. For calculation of total sleep time, total NREM sleep time, and total REM sleep time, data were averaged from 2 consecutive days.

The time course of delta power (1–4 Hz) in NREM sleep was computed as previously described (5–8). To investigate the temporal progression of delta power, values were computed relative to the last 4 hrs of the light phase (i.e., ZT 8 to 12 defined as 1) in baseline. This interval corresponds to the period with lowest delta power (9, 10). These analyses were restricted to the light phase, because in agreement with previous mouse studies (9, 11), there was too little NREM sleep in most mice during the active (dark) phase for accurate assessments. Relative EEG power spectra analysis of each state was conducted by calculating the percentage of each frequency bin to total 1–30 Hz of EEG signals during that state. Absolute EEG power data were also presented as supplementary information to the relative EEG power data.

Fiber photometry. The fiber photometry experiment and data analysis were performed as previously described (1). Nps-Cre mice were infused with AAV1/Syn-Flex-GCaMP6s-WPRE-SV40

virus (Addgene, Cat # 100833-AAV1, RRID: Addgene_100833) (150-300 nL) unilaterally into the peri-LC, PB, and CGPn using the coordinates described above. Two weeks after virus injection, mice were implanted with a 400-μm mono fiber-optic cannula (Doric Lenses) directly above the peri-LC at -5.55 mm (AP); +0.7 mm (ML); -3.7 mm (DV); the PB at -4.9 mm (AP); +1.7 mm (ML); -3.8 mm (DV); and the CGPn at -5.6 mm (AP); 0.0 mm (ML); -4.5 mm (DV). Cannulas were secured to the skull using a base layer of SuperGlue (LocTite) followed by a second layer of dental cement (Ortho-Jet; Lang, Wheeling, IL). EEG/EMG headsets were installed after cannula implantation. Behavioral experiments were conducted 2 weeks later to allow for sufficient recovery and virus expression. All animals were allowed to recover after patch cord attachment for at least 3 days.

A rig for performing fiber photometry recordings was constructed following basic specifications previously described with modifications (12, 13). Briefly, a 473-nm LED (ThorLabs) was used as the GCaMP6s excitation source. A 405-nm LED was used as the isosbestic control. Both LEDs were fed into a fluorescence mini-cube (Doric Lenses, Quebec) that reflected the LED signal through two patch cords into the mouse cannula. Commutators were placed between the patch cords, which allowed the animals to move freely during recording. Fluorescence signals were then reflected back from the mini-cube to a photoreceiver (Newport 2151). The signal was output into a RZ5P Processor (Tucker-Davis Technologies), which allowed for real-time modulation and demodulation of the signal.

All fiber photometry data were subjected to the following minimal processing steps in MATLAB. dF/F indicates (recorded fluorescence-F_0)/F_0. For calculation of dF/F in the spontaneous sleep/wake state, trials were randomly started during the daytime and lasted for 2–3 hrs. F_0 was defined as the median fluorescence over the trial. Different states were determined based on EEG/EMG scoring. The dF/F value for each state was then calculated by averaging the value (dF/F ^{473}-dF/F ^{405}) from the first 10–15 episodes of that state for each mouse.

Electrophysiological recording. As previously described (2), mice were euthanized with 4% isoflurane, perfused with ice-cold sucrose cutting solution (containing [in mM]: 228 sucrose, 26 $NaHCO_3$, 11 glucose, 2.5 KCl, 1 NaH2PO_4, 7 $MgSO_4$ and 0.5 $CaCl_2$, equilibrated with 95% O_2 and 5% CO_2, pH 7.4), and decapitated. Brain slices (250 μm thick) were sectioned with a vibratome (LEICA VT1200S) in cutting solution and recovered for 1 hr in artificial cerebrospinal fluid (ACSF) containing (in mM): 119 NaCl, 26 $NaHCO_3$, 11 glucose, 2.5 KCl, 1 NaH_2PO_4, 1.3 $MgSO_4$, and 2.5 $CaCl_2$.

The recording pipette was pulled from borosilicate glass capillaries (BF150-110-10HP, Sutter Instrument), had a resistance in the range of 3–6 MΩ, and was tip filled with internal solution before being backfilled with internal solution. The internal solution consisted of (in mM): 135 K-gluconate, 7 KCl, 4 Mg-ATP, 0.4 Na_2-GTP, 10 HEPES, 0.5 EGTA, and 10 phosphocreatine (295 mOsm/L, pH 7.25). For validation of hM3D(Gq)'s function, current-clamp recording of neurons was performed under guidance of mCherry expression visualized through a fluorescent microscope (BX51, Olympus). Neurons with stable membrane potentials for at least 2 min were bath applied with CNO (30 μM) for 2–3 min and then washed with ACSF. For recording of light-evoked post-synaptic currents, voltage-clamp recording of neurons was performed under a 40X objective of infrared differential interference contrast microscopy (BX51, Olympus). Light (473 nm, Sutter Instrument) pulses were delivered through a 40X objective. For test of modification of synaptic inputs by NPS application, voltage-clamp recording of neurons was performed under guidance of GFP expression. Neurons were bath applied with NPS (30 μM) for 3 min and then washed with ACSF. Recording was made with a patch-clamp amplifier (MultiClamp 700B, Axon Instruments), and signals were filtered at 1 kHz and sampled at 10 kHz using Digidata 1550B (Axon Instruments). Recordings were analyzed using Clampfit (Axon instruments) or custom-written software based on MATLAB (Mathworks). The data were discarded if the series resistance varied by >20% during recording. All drugs were purchased from Sigma-Aldrich unless otherwise mentioned.

Anterograde and retrograde tracing. To trace the axonal projections of NPS^+ neurons, *Nps*-Cre mice were infused with AAV-EF1a-DIO-hChR2(H134R)-EYFP-WPRE-HGHpA virus (Addgene, Cat

20298-AAV5, RRID: Addgene_20298). Mice were sacrificed 3–5 weeks later for histological analysis.

For rabies-mediated retrograde trans-synaptic tracing, experiments were carried out as described (14). Briefly, 300 nl of mixed AAV1-syn-FLEX-splitTVA-EGFP-tTA (1:200 dilution, Addgene, Cat # 100798-AAV1, RRID: Addgene_100798) and AAV1-TREtight-mTagBFP2-B19G (1:20 dilution, Addgene, Cat # 100799-AAV1, RRID: Addgene_ 100799) was injected into the target regions of the *Nps*-Cre mice. One week later, EnvA G-Deleted Rabies-tdTomato (Salk) was injected into the same region, and mice were sacrificed 7 days later for histological analysis.

Histology and immunohistochemistry. As previously described (1), mice were transcardially perfused with phosphate-buffered saline (PBS) followed by 4% paraformaldehyde (PFA) in PBS. Brains were postfixed overnight in 4% PFA and placed in 30% sucrose for another 24 hrs. Free-floating sections (40–50 μm) were prepared with a cryostat (Leica). For EGFP, GCaMP6s, and DREADDs verification, slices were immediately mounted onto glass slides with DAPI mounting media, cover-slipped, and imaged.

For TeNT verification, sections were washed three times in PBS and then blocked for 1 hr in superblock buffer (Thermo Fisher Scientific). The sections were then incubated with a primary antibody for GFP (1/1,000 rabbit anti GFP, Thermo Fisher Scientific, Cat # A1112, RRID: AB_221569) at 4°C overnight. After washing three times in Tris-buffered saline containing Tween 20 (TBST), the sections were incubated with corresponding secondary antibodies (Jackson Immuno Research) for another 2 hrs at room temperature before being mounted onto glass slides.

In situ hybridization (ISH) of *Nps and Gfp* was performed using the RNAscope assay (Cat # 323110, Advanced Cell Diagnostics, Multiplex Fluorescent Detection Reagents v2). Nps-Cre mice were perfused as described above. Brains were postfixed overnight in 4% PFA and then immersed in 20% sucrose, followed by 30% sucrose, and brains sank to the bottom of the container during each immersion. Brains were embedded in cryo-embedding medium and rapidly frozen in -80°C freezer. Then 40-μm frozen sections were obtained, and all subsequent steps were performed according to the manufacturer's instructions. Probes used: Mm-Nps (485201), EGFP (400281-C2). The slides were imaged as described below.

Fluorescence imaging. Images were acquired on an inverted Nikon Ti microscope (Nikon) using NIS-Element software (Nikon), equipped with a Sutter Lambda LS-2 xenon arc lamp, Nikon DS-Qi2 monochrome camera, dichroic FF408/504/581/667/762-Di01 (Semrock). For the imaging of *in situ* hybridization (ISH) sections, a Plan Apo 20×/0.75 objective (Nikon) and excitation/emission filters for FITC (FF02-485/20, FF01-525/30) and Cy3 (FF01-560/25 and FF01-607/36, Semrock) were used. Other fluorescence images were acquired using the same microscope with 10X/0.45 NA objective (Nikon).

Whole-brain tissue section images were taken by stitching images from over 50 xy-positions with an xy-pixel size of 1.36 μm/px. Raw images were processed with NIH ImageJ software. All microscope images for this study were acquired at the Center for Advanced Light Microscope at UCSF.

CasRx AAV vector. The CasRx AAV vector was modified from a previous report (15). Briefly, the CasRx ORF cassette was inserted into an AAV/syn-DIO backbone. This design ensured expression of CasRx under the synapsin (Syn) promoter in a Cre-dependent manner. The U6-CasRx-gRNA cassette, including two complementary 30-bp sequences (below) against target mRNA (*Nps*), was further inserted after the Syn-DIO-CasRx to generate the Syn-DIO-CasRx-U6-Nps gRNA (CasRx-Nps). Syn-DIO-CasRx (CasRx-only) without the gRNA cassette was used as a control. AAV8s were produced by Vigene Bioscience using an AAV packaging service with a typical titer around 10^13 GC/ml.
Nps (30-bp sequence)
gRNA1: CGACTCCGTTGCGAAAGGACCTTTTCATCG

gRNA2: AAGAGAGGACCGGATAACACCAAAGCACGT

Supplemental Figures

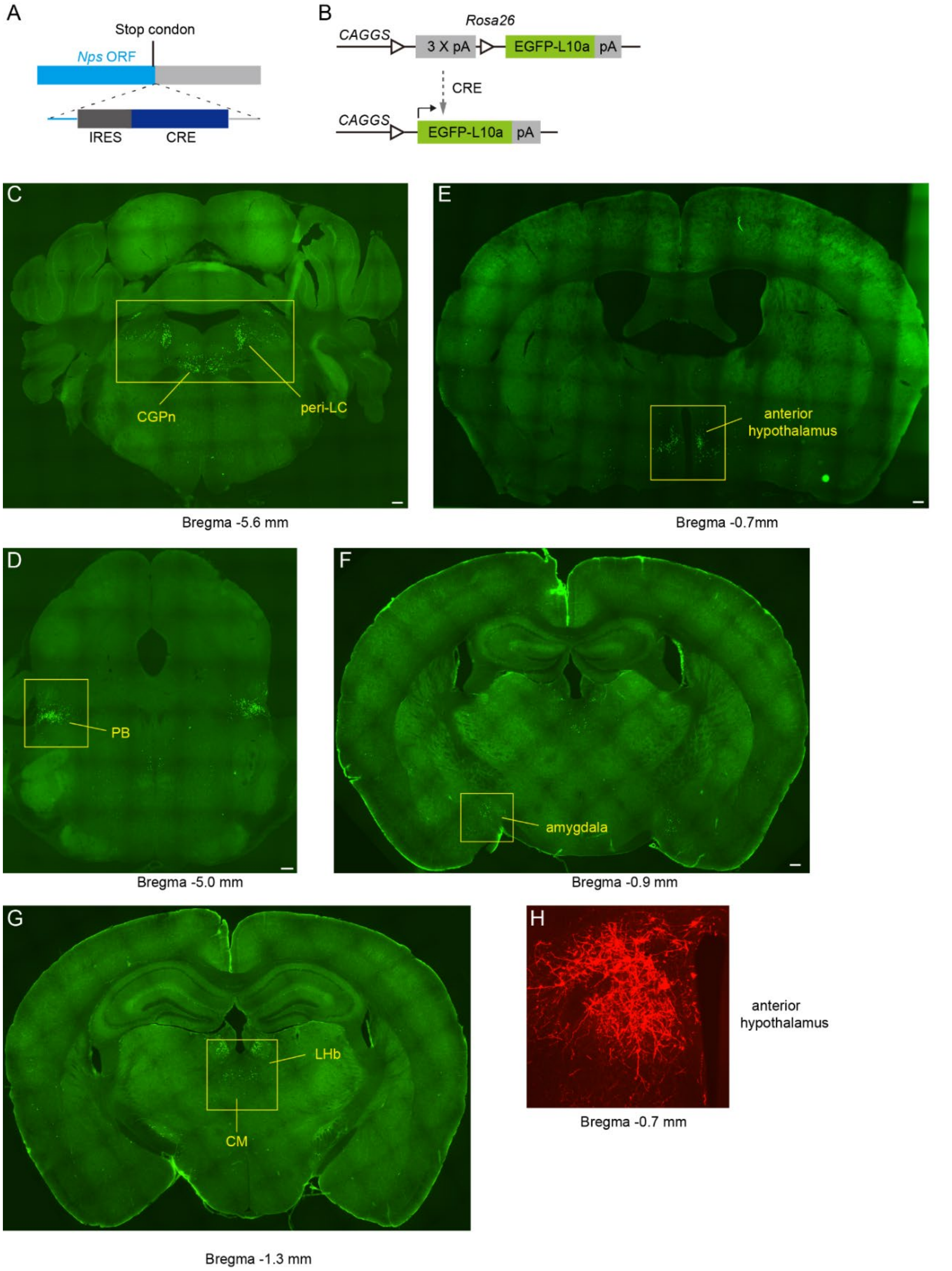

Fig. S1. Potential NPS⁺ cells in the intact coronal brain sections. (*A*) *Nps*-ires-Cre mice generated by CRISPR-mediated recombination. (*B*) Schematic of *Rosa26*-EGFP-L10a allele and experimental workflow. Following CRE-mediated removal of the transcriptional stop cassette (3x

pA), EGFP-L10a expression is driven by the CAG promoter. (*C–G*) Representative brain sections from *Nps*-Cre; EGFP-L10a mouse showing GFP^+ cells in the peri-LC & CGPn (*C*), PB (*D*), anterior hypothalamus (*E*), amygdala (*F*) and LHb & CM (*G*). (*H*) Representative brain section of anterior hypothalamus from *Nps*-Cre mouse which was injected with AAV8/hSyn-DIO-mCherry.

LC, locus coeruleus; PB, parabrachial nucleus; CGPn, central gray of the pons; LHb, lateral habenular nucleus; CM, central medial nucleus of thalamus.

Representative brain sections from n = 3 mice. Scale bar, 200 μm.

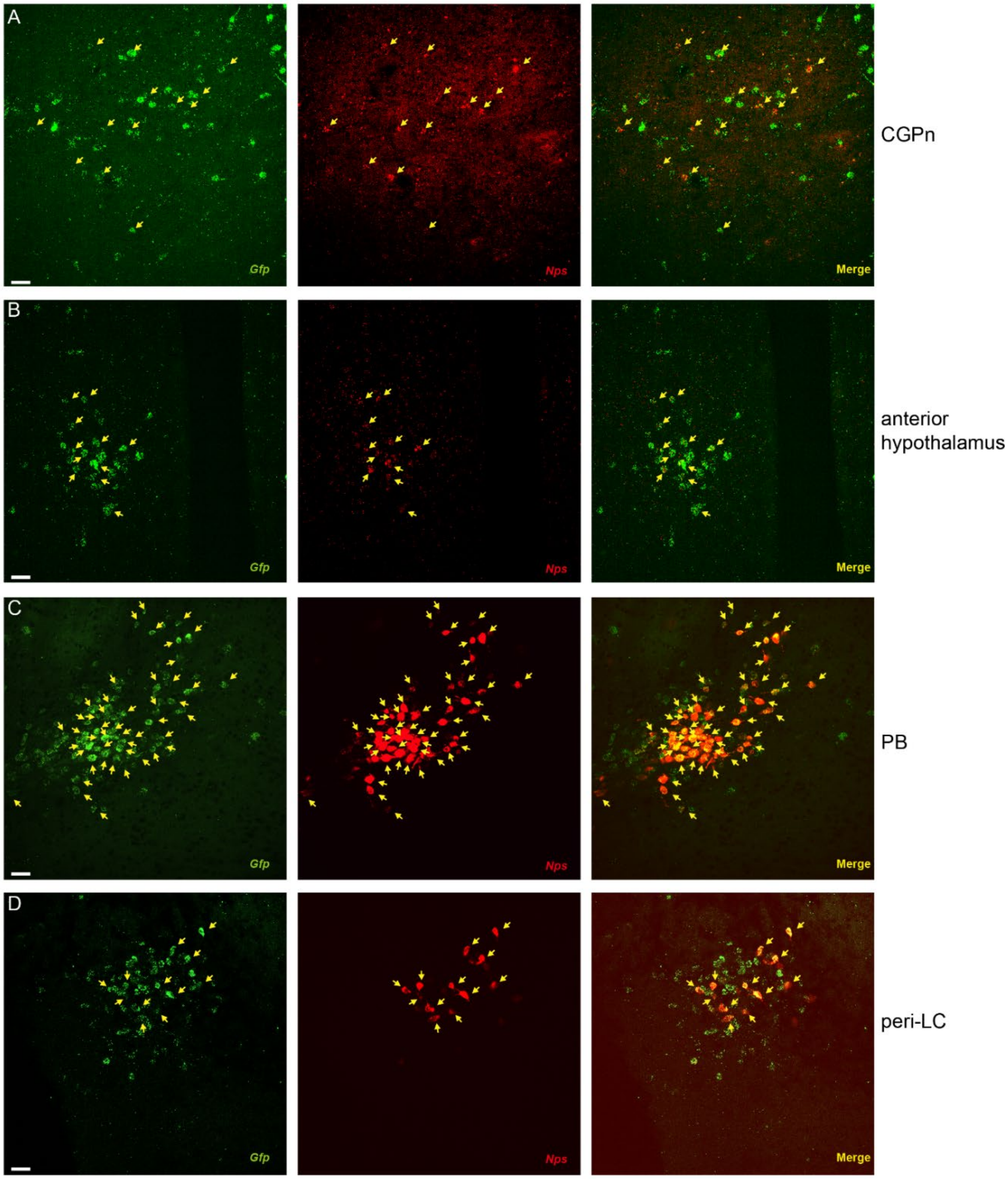

Fig. S2. Co-localization of *Gfp* and *Nps.* (*A*–*D*) Representative brain sections from *Nps*-Cre; EGFP-L10a showing co-localization of *Gfp* (green) with *Nps* mRNA (red) in CGPn (*A*), anterior hypothalamus (*B*), PB (*C*) and peri-LC (*D*). Merged images are shown on the right column (yellow). Arrowheads indicate cells in which coexpression was detected. Scale bars, 30 µm. n = 4 mice.

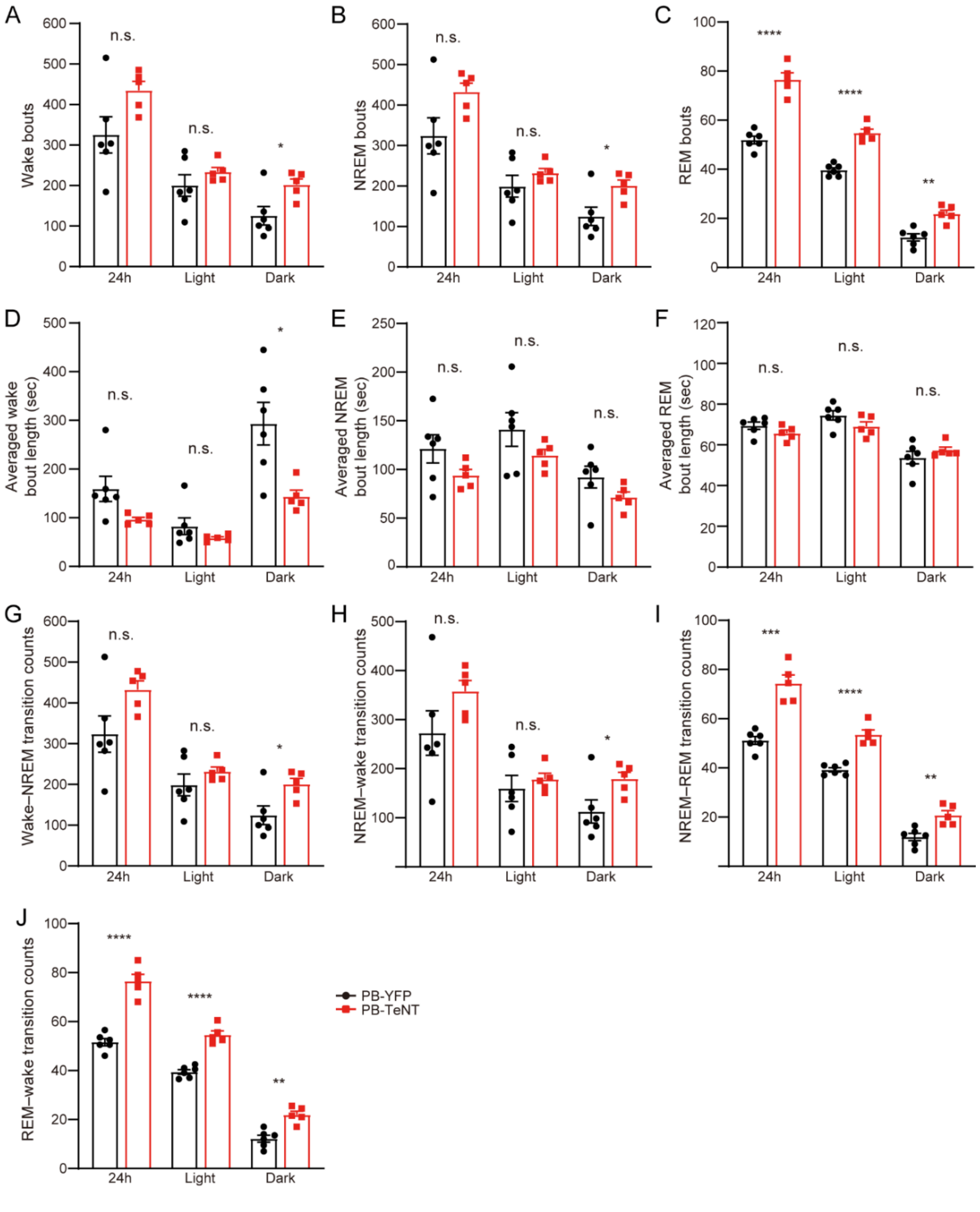

Fig. S3. Sleep/wake fragmentation and transitions of PB-TeNT mice. (*A*–*C*) Wake (*A*), NREM sleep (*B*) and REM sleep (*C*) bouts during 24 hours, light phase and dark phase were calculated in YFP (n = 6) and TeNT (n = 5) injected mice. (*D*–*F*) Average wake (*D*), NREM sleep (*E*) and REM sleep (*F*) episode length during 24 hours, light phase and dark phase were calculated in YFP (n = 6) and TeNT (n = 5) injected mice. (*G*–*J*) Number of transitions of wake–NREM (*G*), NREM–wake (*H*), NREM–REM (*I*) and REM–wake (*J*) during 24 hours, light phase and dark phase were calculated in YFP (n = 6) and TeNT (n = 5) injected mice. *$P < 0.05$, **$P < 0.01$, ***$P < 0.001$, ****$P < 0.0001$, n.s. = not significant. Two-tailed Student's *t*-test for (*A*–*J*). Error bars represent ± SEM.

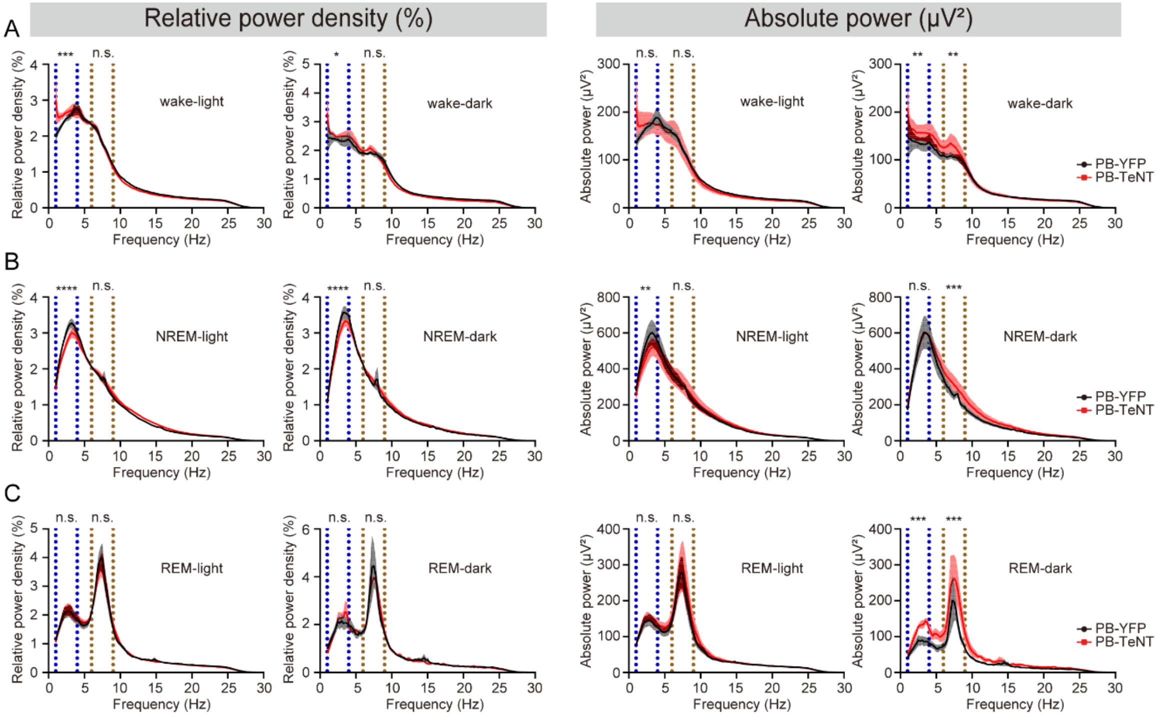

Fig. S4. Spectral composition of PB-TeNT mice. (*A*) Spectral composition of YFP (n = 6) and TeNT (n = 5) injected mice during wakefulness. (*B*) Spectral composition of YFP (n = 6) and TeNT (n = 5) injected mice during NREM sleep. (*C*) Spectral composition of YFP (n = 6) and TeNT (n = 5) injected mice during REM. *P < 0.05, **P < 0.05, ***P < 0.001, ****P < 0.0001, n.s. = not significant. Ordinary two-way ANOVA for (*A–C*). Delta frequency (1–4 Hz) is indicated within two dashed blue lines, and theta frequency (6–9 Hz) is within dashed brown lines. Absolute power data (right panel) were presented as supplementary information to the relative power density data (left panel). Shaded area around the corresponding curve represents ± SEM.

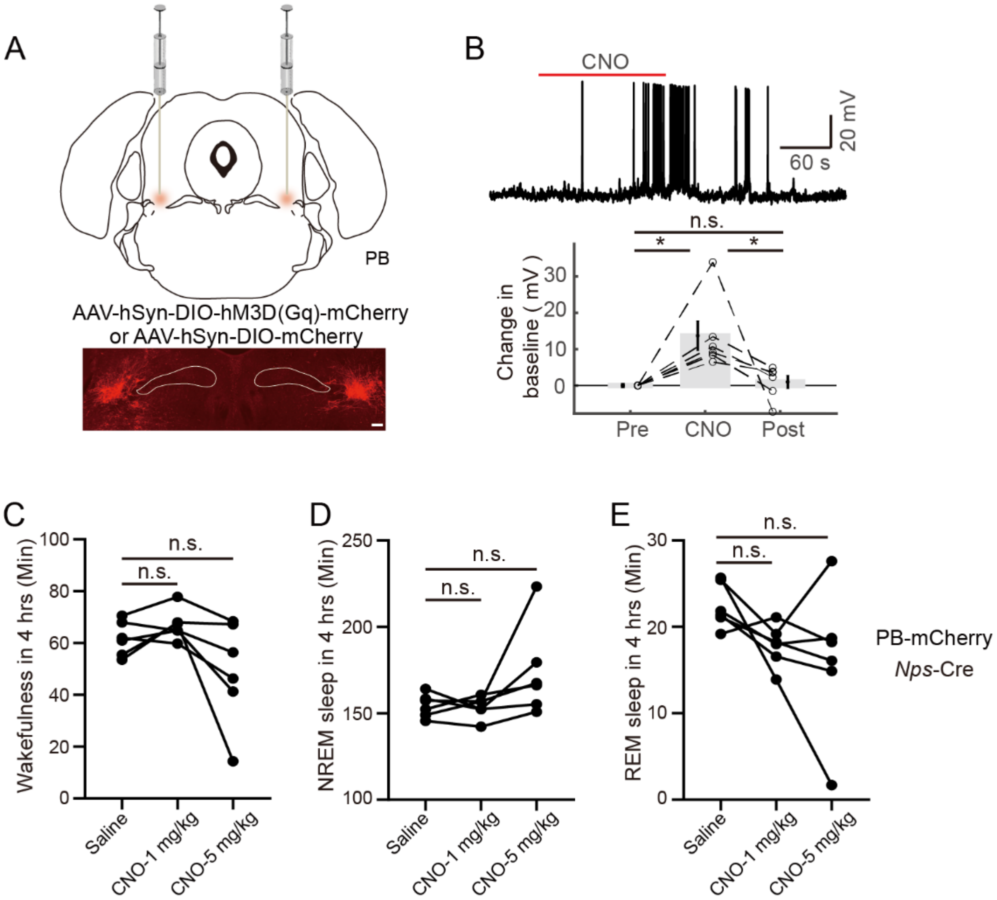

Fig. S5. DREADDs experiments on PB NPS⁺ neurons. (*A*) Schematic slice of chemogenetic activation of PB NPS⁺ neurons. (*B*) Top: Representative voltage trace from one *Nps*-Cre neuron expressing hM3D(Gq)-mCherry for activation. Red bar indicates CNO perfusion window. Bottom: Summary data for membrane potential changes before, during and after perfusion of brain slices with CNO (30 μM). Each dashed line with connected symbols indicates one neuron before, during and after CNO. n = 6 neurons. (*C–E*) Quantified results for total wakefulness (*C*), NREM (*D*), and REM (*E*) sleep recorded 4 hours after saline or CNO injection at ZT6 in the mCherry (n = 6) injected mice (PB). *P < 0.05, n.s. = not significant. RM one-way ANOVA, post-hoc Dunnett's multiple comparisons test for (C–*E*). Wilcoxon signed rank test for *B*. Error bars represent ± SEM. Scale bar, 200 μm.

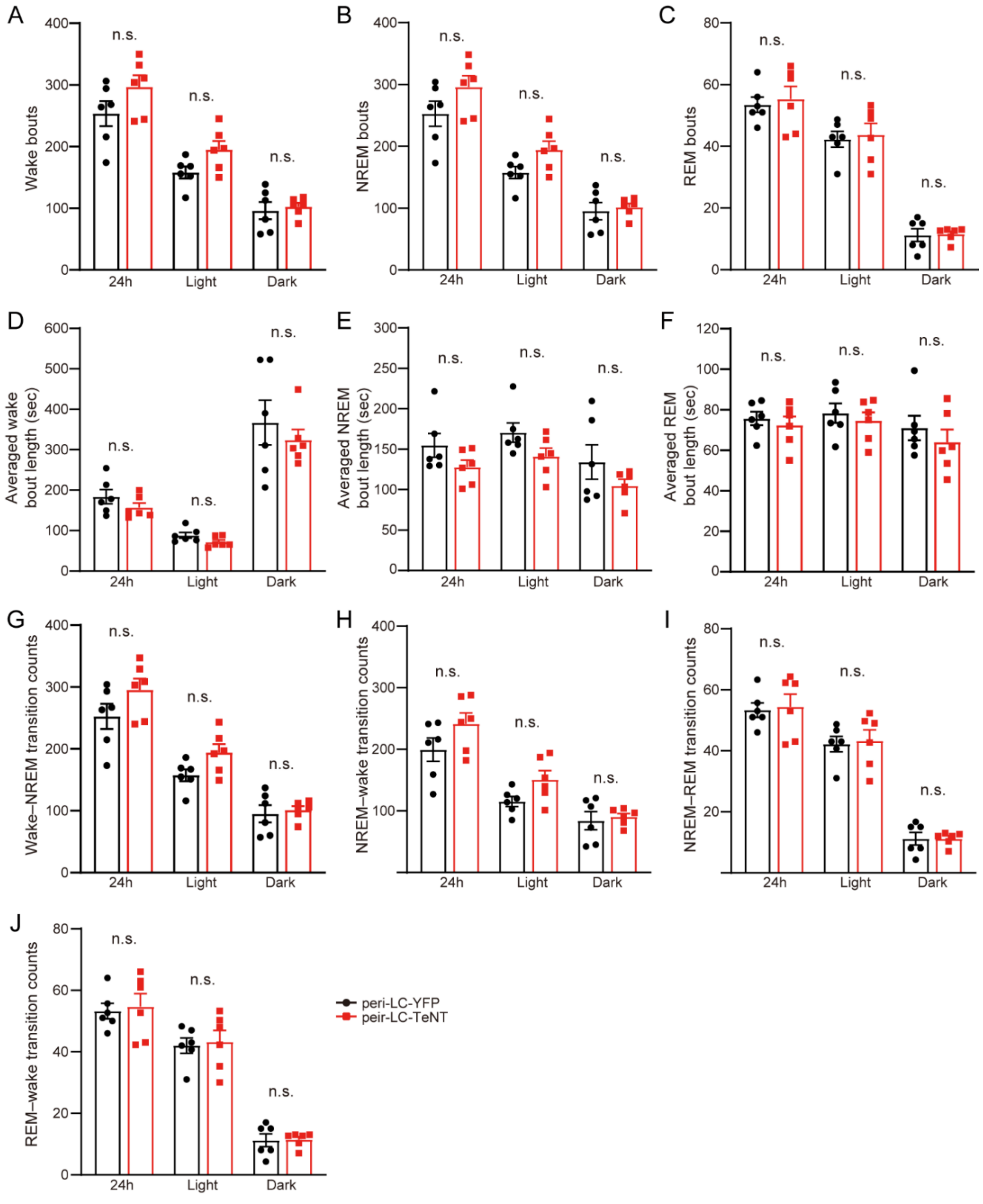

Fig. S6. Sleep/wake fragmentation and transitions of peri-LC-TeNT mice. (*A–C*) Wake (*A*), NREM sleep (*B*) and REM sleep (*C*) bouts during 24 hours, light phase and dark phase were calculated in YFP (n = 6) and TeNT (n = 6) injected mice. (*D–F*) Average wake (*D*), NREM sleep (*E*) and REM sleep (*F*) episode length during 24 hours, light phase and dark phase were calculated in YFP (n = 6) and TeNT (n = 6) injected mice. (*G–J*) Number of transitions of wake–NREM (*G*), NREM–wake (*H*), NREM–REM (*I*) and REM–wake (*J*) during 24 hours, light phase and dark phase were calculated in YFP (n = 6) and TeNT (n = 5) injected mice. n.s. = not significant. Two-tailed Student's *t*-test for (*A–J*). Error bars represent ± SEM.

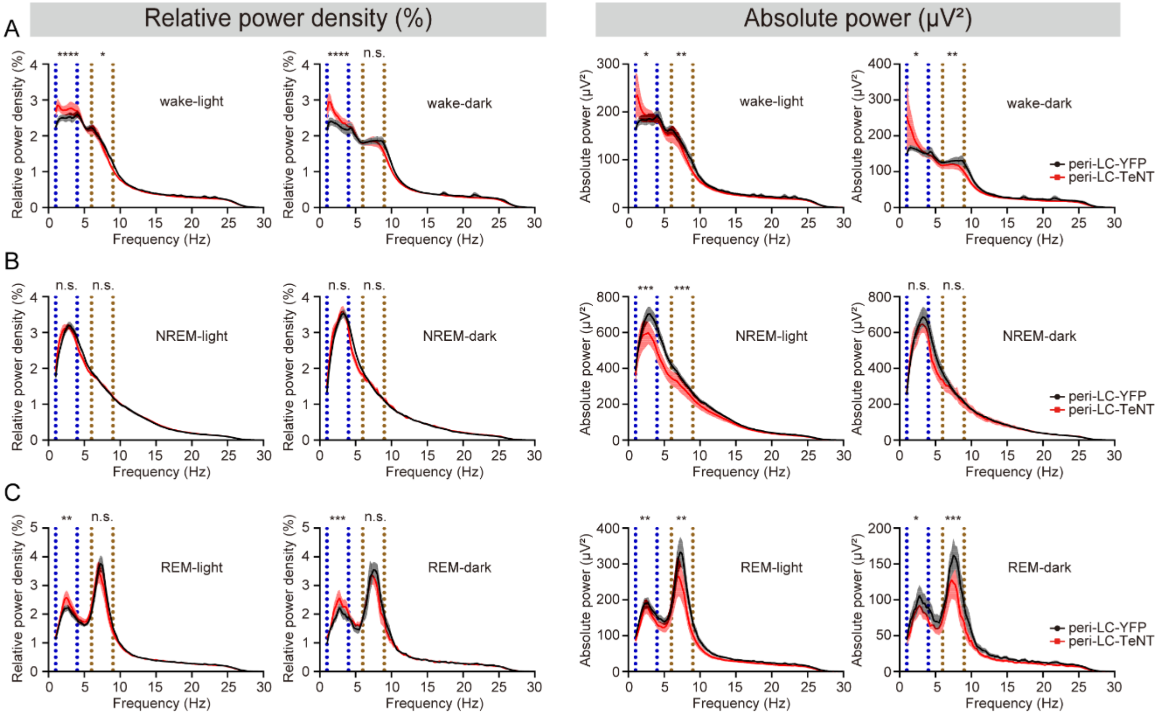

Fig. S7. Spectral composition of peri-LC-TeNT mice. (*A*) Spectral composition of YFP (n = 6) and TeNT (n = 6) injected mice during wakefulness. (*B*) Spectral composition of YFP (n = 6) and TeNT (n = 6) injected mice during NREM sleep. (*C*) Spectral composition of YFP (n = 6) and TeNT (n = 6) injected mice during REM. **P* < 0.05, ***P* < 0.01, ****P* < 0.001, *****P* < 0.0001, n.s. = not significant. Ordinary two-way ANOVA for (*A*–*C*). Delta frequency (1–4 Hz) is indicated within two dashed blue lines, and theta frequency (6–9 Hz) is within dashed brown lines. Absolute power data (right panel) were presented as supplementary information to the relative power density data (left panel). Shaded area around the corresponding curve represents ± SEM.

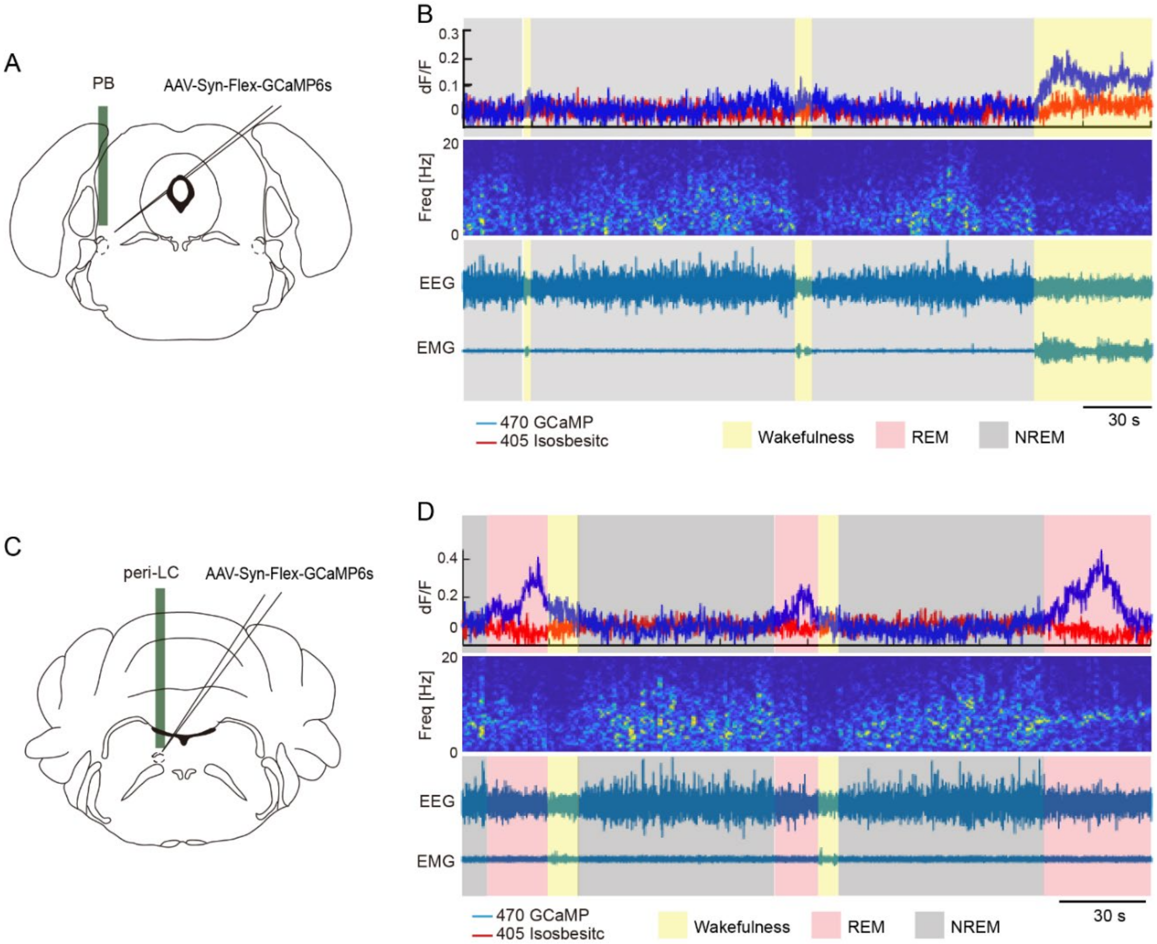

Fig. S8. Fiber photometry recording of PB and peri-LC NPS⁺ cells. (*A*) Schematic showing delivery of the GCaMP expressing virus and the placement of the fiber tip above PB. (*B*) Representative EEG power spectrogram, EEG, EMG, and fluorescence traces across spontaneous sleep/wake states of PB fiber photometry recording. (*C*) Schematic showing delivery of the GCaMP expressing virus and the placement of the fiber tip above peri-LC. (*D*) Representative EEG power spectrogram, EEG, EMG, and fluorescence traces across spontaneous sleep/wake states of peri-LC fiber photometry recording.

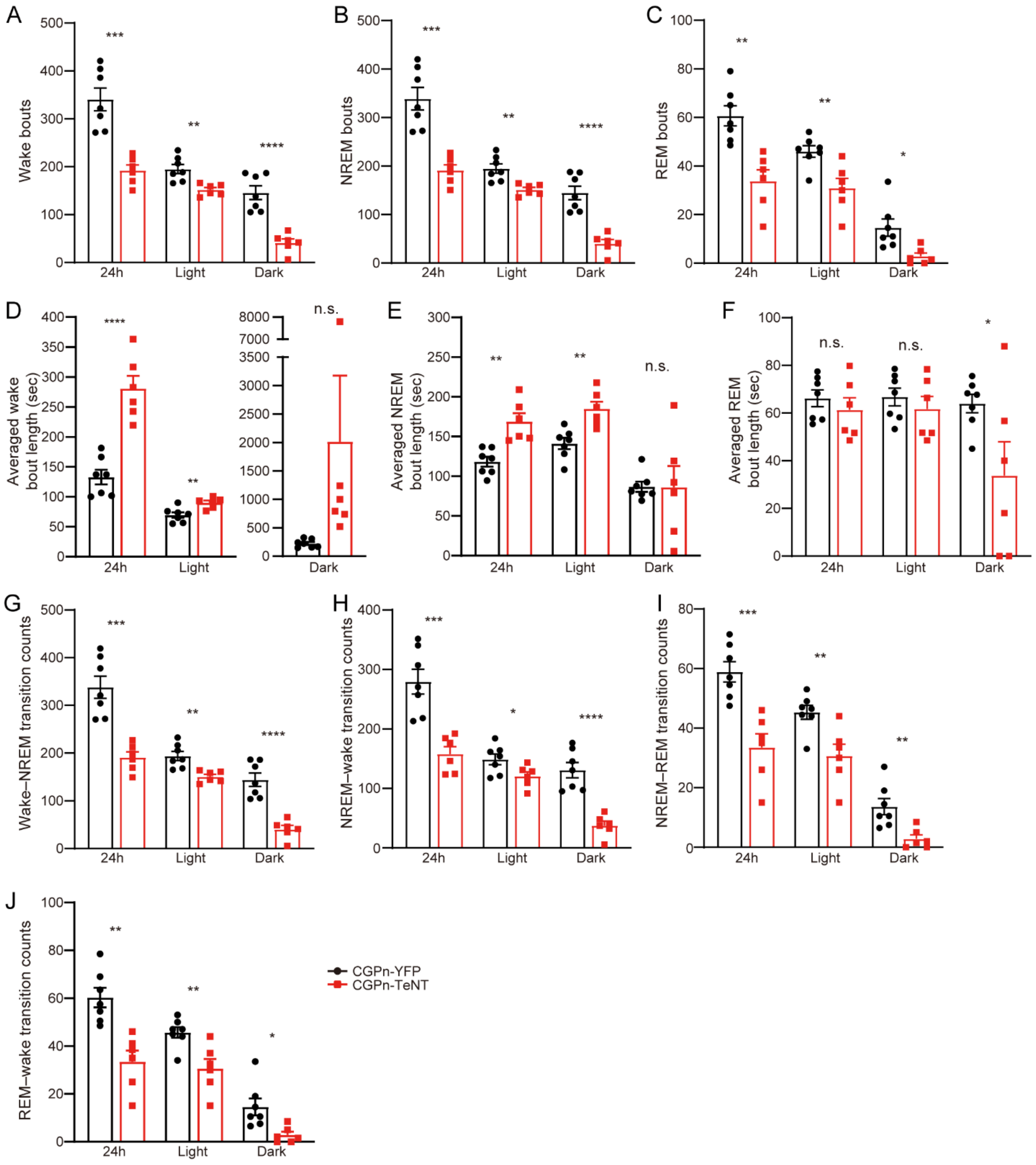

Fig. S9. Sleep/wake fragmentation and transitions of CGPn-TeNT mice. (*A*–*C*) Wake (*A*), NREM sleep (*B*) and REM sleep (*C*) bouts during 24 hours, light phase and dark phase were calculated in YFP (n = 7) and TeNT (n = 6) injected mice. (*D*–*F*) Average wake (*D*), NREM sleep (*E*) and REM sleep (*F*) episode length during 24 hours, light phase and dark phase were calculated in YFP (n = 7) and TeNT (n = 6) injected mice. (*G*–*J*) Number of transitions of wake–NREM (*G*), NREM–wake (*H*), NREM–REM (*I*) and REM–wake (*J*) during 24 hours, light phase and dark phase were calculated in YFP (n = 7) and TeNT (n = 5) injected mice. **P* < 0.05, ***P* < 0.01, ****P* < 0.001, *****P* < 0.0001, n.s. = not significant. Two-tailed Student's *t*-test for (*A*–*J*). Error bars represent ± SEM.

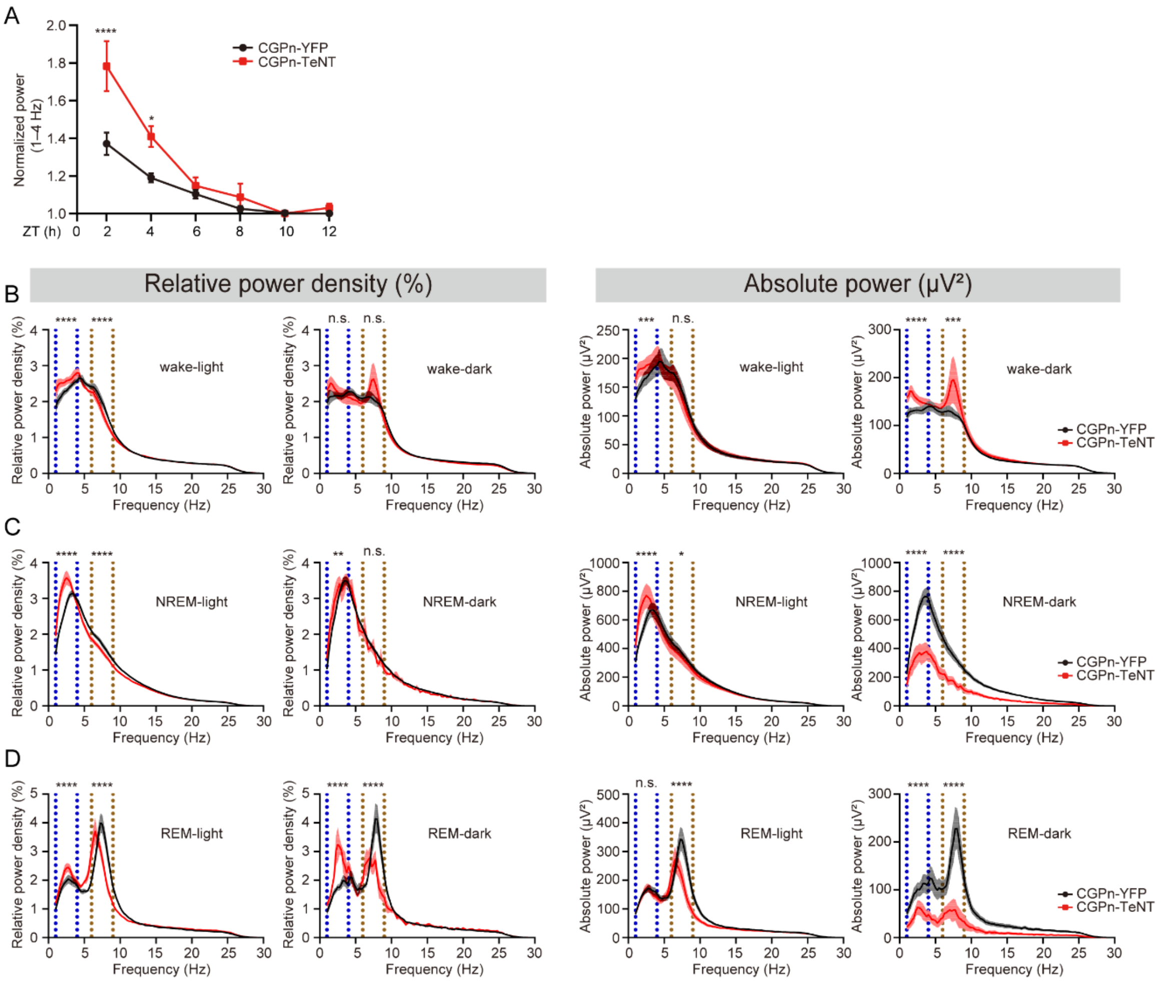

Fig. S10. Spectral composition of CGPn-TeNT mice. (*A*) Time course of EEG delta power (1–4 Hz) during NREM sleep across the light phase for YFP (n = 7) and TeNT (n = 6) injected mice. (*B*) Spectral composition of YFP (n = 7) and TeNT (n = 5) injected mice during wakefulness. (*C*) Spectral composition of YFP (n = 7) and TeNT (n = 6) injected mice during NREM sleep. (*D*) Spectral composition of YFP (n = 7) and TeNT (n = 6) injected mice during REM. *$P < 0.05$, **$P < 0.01$, ***$P < 0.001$, ****$P < 0.0001$, n.s. = not significant. Two-way ANOVA, post-hoc Sidak's multiple comparisons test for (*A*). Ordinary two-way ANOVA for (*B*–*D*). Delta frequency (1–4 Hz) is indicated within two dashed blue lines, and theta frequency (6–9 Hz) is within dashed brown lines (*B*–*D*). Absolute power data (right panel) were presented as supplementary information to the relative power density data (left panel) (*B*–*D*). Shaded area around the corresponding curve represents ± SEM.

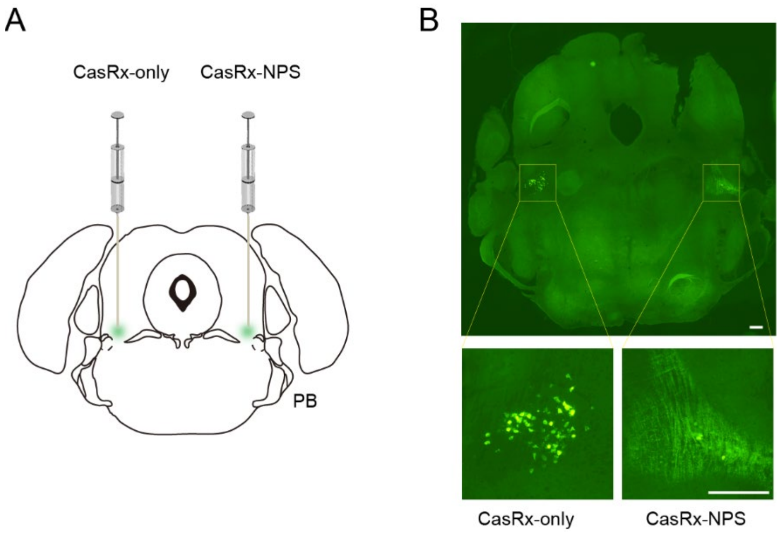

Fig. S11. Controls for *Nps* knockdown. Representative *Nps* mRNA detected by *in situ* hybridization in the PB region after CasRx-only (left side) or CasRx-NPS (right side) virus delivery. Magnified box areas shown on the bottom. Scale bars, 200 µm.

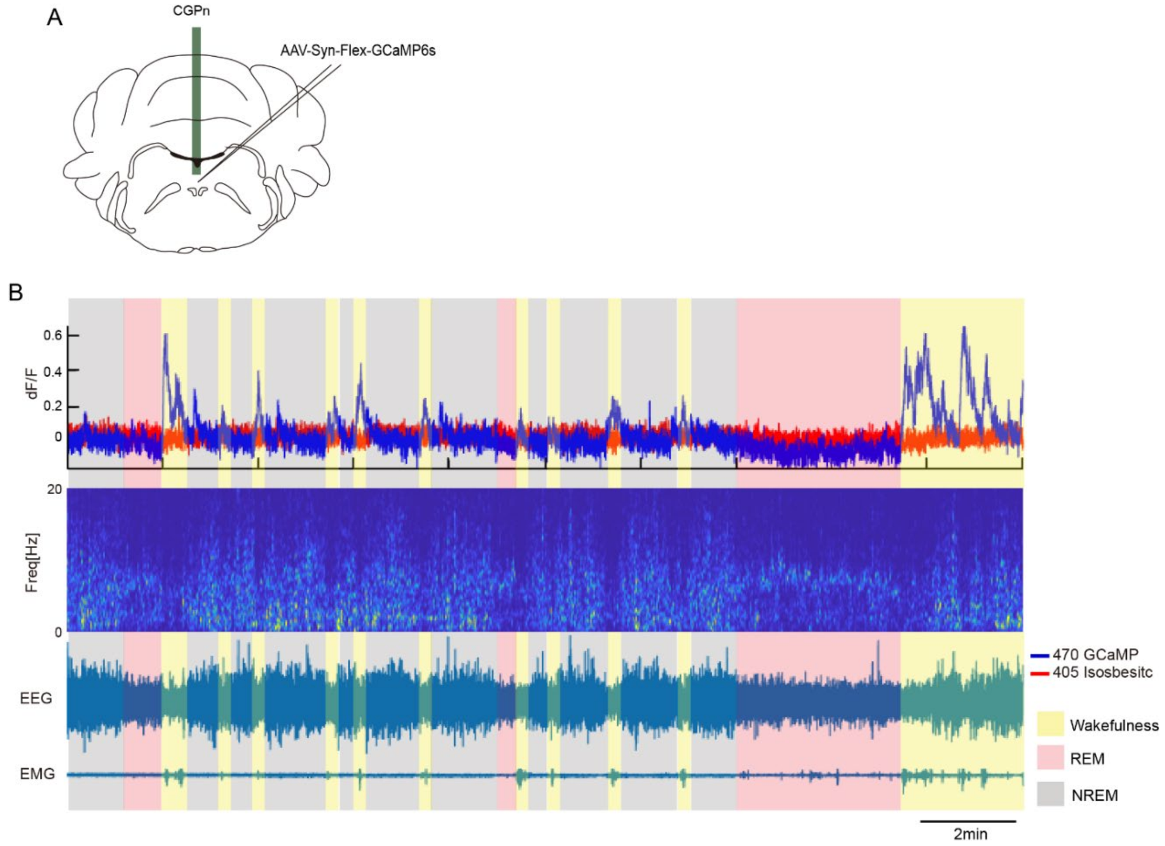

Fig. S12. Fiber photometry recording of CGPn NPS+ cells. (*A*) Schematic showing delivery of the GCaMP-expressing virus and placement of the fiber tip above the CGPn. (*B*) Representative EEG power spectrogram, EMG and fluorescence traces across spontaneous sleep/wake states.

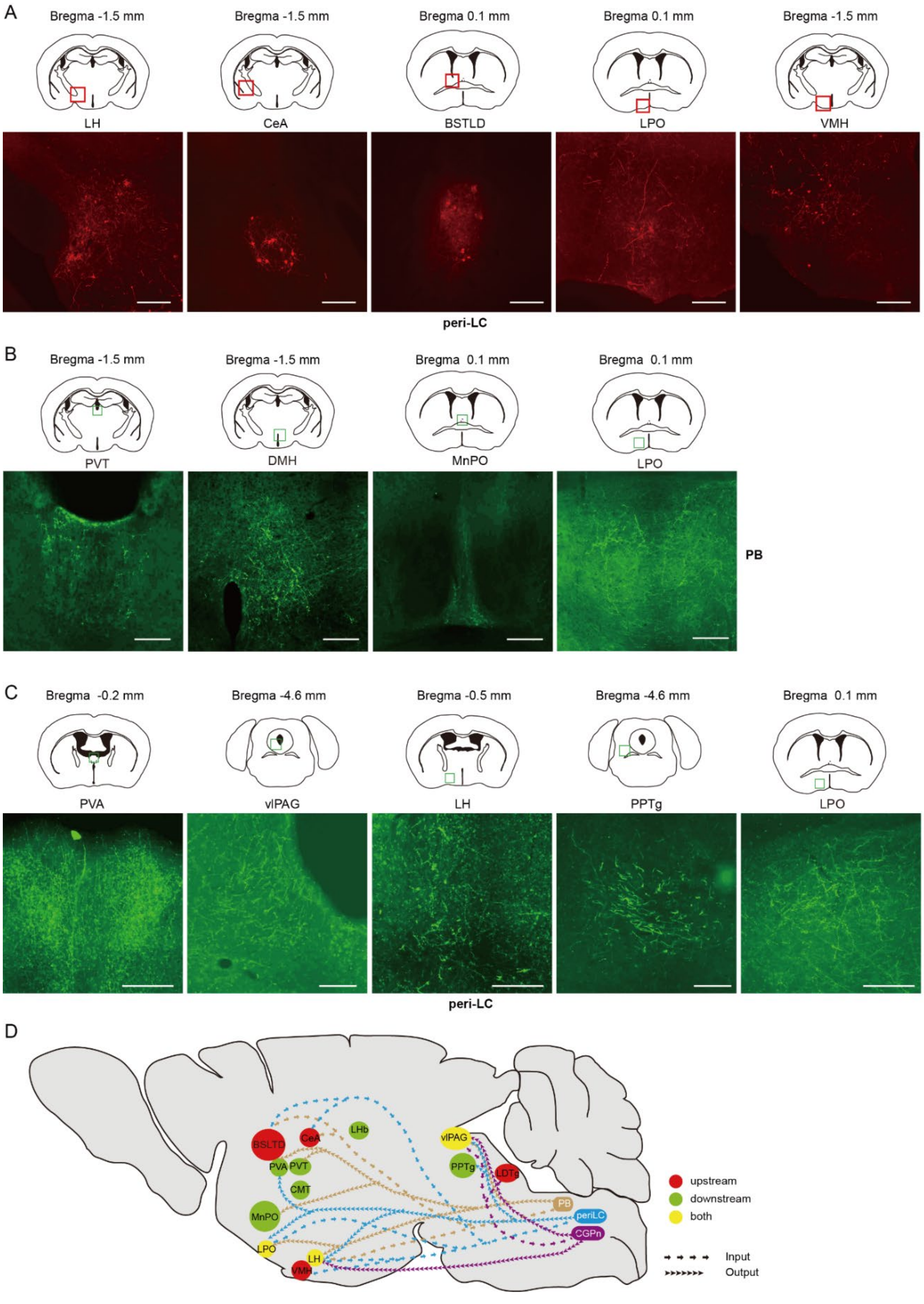

Fig. S13. Upstream and downstream nuclei of NPS⁺ cells. (*A*) RV-labeled presynaptic neurons (red squares) of peri-LC NPS⁺ cells. (*B*) Expression of eYFP in some PB NPS⁺ neuron terminals at

indicated regions (green squares). (*C*) Expression of eYFP in peri-LC NPS⁺ neuron terminals at indicated regions (green squares).

BSTLD, laterodorsal bed nucleus of the stria terminalis; CeA, central amygdala; DMH, dorsomedial hypothalamus; LH, lateral hypothalamus; LPO, lateral preoptic area; MnPO, the median preoptic nucleus; PPTg, pedunculopontine tegmental nucleus; PVA, anterior nucleus of paraventricular thalamus; PVT, paraventricular thalamic nucleus; vlPAG, ventrolateral periaqueductal gray; VMH, ventromedial hypothalamus.

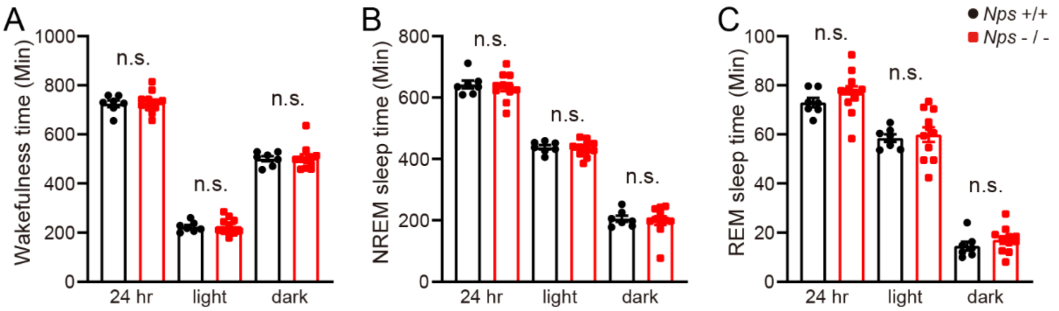

Fig. S14. Knockout of *Nps* does not change the vigilance state percentage. (*A–C*) Total wakefulness time (*A*), NREM sleep time (*B*), and REM sleep time (*C*) by EEG/EMG within 24 hours, light phase and dark phase were calculated in *Nps* +/+ (n = 7) and *Nps* -/- (n = 11) mice. n.s.=not significant. Two-tailed Student's *t*-test for (*A–C*).

SI References

1. G. Shi, *et al.*, A Rare Mutation of β1-Adrenergic Receptor Affects Sleep/Wake Behaviors. *Neuron* (2019) https:/doi.org/10.1016/j.neuron.2019.07.026.
2. G. Shi, *et al.*, Mutations in Metabotropic Glutamate Receptor 1 Contribute to Natural Short Sleep Trait. *Curr. Biol.* (2020) https:/doi.org/10.1016/j.cub.2020.09.071.
3. L. Xing, *et al.*, Mutant neuropeptide S receptor reduces sleep duration with preserved memory consolidation. *Sci. Transl. Med.* **11** (2019).
4. C. Anaclet, *et al.*, The GABAergic parafacial zone is a medullary slow wave sleep-promoting center. *Nat. Neurosci.* **17**, 1217–1224 (2014).
5. C. Cirelli, Locus Ceruleus Control of Slow-Wave Homeostasis. *J. Neurosci.* **25**, 4503–4511 (2005).
6. P. Franken, *et al.*, NPAS2 as a transcriptional regulator of non-rapid eye movement sleep: Genotype and sex interactions. *Proc. Natl. Acad. Sci.* **103**, 7118–7123 (2006).
7. A. Vassalli, P. Franken, Hypocretin (orexin) is critical in sustaining theta/gamma-rich waking behaviors that drive sleep need. *Proc. Natl. Acad. Sci.* **114**, E5464–E5473 (2017).
8. Z. Wang, *et al.*, Quantitative phosphoproteomic analysis of the molecular substrates of sleep need. *Nature* (2018) https:/doi.org/10.1038/s41586-018-0218-8.
9. P. Franken, D. Chollet, M. Tafti, The homeostatic regulation of sleep need is under genetic control. *J. Neurosci.* **21**, 2610–2621 (2001).
10. G. M. Mang, P. Franken, "Sleep and EEG Phenotyping in Mice" in *Current Protocols in Mouse Biology*, (2012) https:/doi.org/10.1002/9780470942390.mo110126.
11. M. M. Halassa, *et al.*, Astrocytic Modulation of Sleep Homeostasis and Cognitive Consequences of Sleep Loss. *Neuron* **61**, 213–219 (2009).
12. L. A. Gunaydin, *et al.*, Natural neural projection dynamics underlying social behavior. *Cell* **157**, 1535–1551 (2014).
13. Y. Chen, Y. C. Lin, T. W. Kuo, Z. A. Knight, Sensory Detection of Food Rapidly Modulates Arcuate Feeding Circuits. *Cell* **160**, 829–841 (2015).
14. T. K. Lavin, L. Jin, N. E. Lea, I. R. Wickersham, Monosynaptic Tracing Success Depends Critically on Helper Virus Concentrations. *Front. Synaptic Neurosci.* (2020) https:/doi.org/10.3389/fnsyn.2020.00006.
15. S. Konermann, *et al.*, Transcriptome Engineering with RNA-Targeting Type VI-D CRISPR Effectors. *Cell* **173**, 665-676.e14 (2018).
